# In Vitro Metabolism of Doping Agents (Stanozolol, LGD-4033, Anastrozole, GW1516, Trimetazidine) by Human Seminal Vesicle and Liver Fractions

**DOI:** 10.3390/metabo15070452

**Published:** 2025-07-04

**Authors:** Johanna Sternberg, Insa Peters, Nana Naumann, Andreas Thomas, Mario Thevis

**Affiliations:** 1 Institute of Biochemistry, Center for Preventive Doping Research, German Sport University Cologne, 50933 Cologne, Germany; j.sternberg@biochem.dshs-koeln.de (J.S.); i.peters@biochem.dshs-koeln.de (I.P.); n.naumann@biochem.dshs-koeln.de (N.N.); a.thomas@biochem.dshs-koeln.de (A.T.); 2 European Monitoring Center for Emerging Doping Agents (EuMoCEDA), 50933 Cologne, Germany; 3 European Monitoring Center for Emerging Doping Agents (EuMoCEDA), 53113 Bonn, Germany

**Keywords:** sports doping, metabolism, in vitro, dPCR, LC-HRAM MS

## Abstract

**Background:** In order to address complex scenarios in anti-doping science, especially in cases where an unintentional exposure of athletes to prohibited substances and a corresponding contamination of doping control samples at the collection event are argued, an understanding of tissue-specific drug metabolism is essential. Hence, in this study, the metabolic capacity of the seminal vesicle using in vitro assays was investigated. **Methods:** The aim was to assess whether selected doping-relevant substances—stanozolol, LGD-4033, GW1516, trimetazidine, and anastrozole—are metabolised in seminal vesicle cellular fractions (SV-S9) and how that metabolism compares to biotransformations induced by human liver S9 fractions (HL-S9). Liquid chromatography coupled to high-resolution/accurate mass spectrometry (LC HRAM MS) enabled the sensitive detection and identification of metabolites, revealing a limited metabolic activity of SV-S9. **Results**: For LGD-4033, GW1516, and trimetazidine, minor metabolic transformations were observed, whereas no metabolites of stanozolol or anastrozole were detected. Gene expression analysis using digital polymerase chain reaction (dPCR) confirmed transcripts of CYP2D6, CYP2E1, and CYP2C9 in SV-S9, though no enzymatic activity was detected. Gene expression and enzymatic activity in CYP3A4 and CYP1A2—major hepatic enzymes—were absent in SV-S9. **Conclusions:** Overall, these pilot study results suggest that the seminal vesicle has only a low capacity for xenobiotic metabolism, which translates into a limited role in the biotransformation of drugs and, hence, the metabolic pattern.

## 1. Introduction

Improvements in anti-doping analysis are vital for meeting the various challenges of uncovering doping practices in sport. However, these improvements, which commonly include increased sensitivities offered by modern analytical methods, have also created the necessity of distinguishing between intentional drug abuse (that occurred long ago) and contamination scenarios, as low levels of a substance are compatible with both scenarios [1]. One of these contamination scenarios can be referred to as ‘intimate contact drug (metabolites) residuals’, which may occur when drugs have been transferred to the athlete via skin contact, saliva, or seminal fluid, thereby triggering an Adverse Analytical Finding (AAF) in the doping control sample [1]. The metabolism of substances can be of particular interest in the detection of intimate contact contamination, as metabolite profiles may differ depending on the route of administration (oral/saliva or dermal) and the excretion matrix (urine or seminal fluid) and can therefore serve as markers [1]. Since it has been shown that various substances can be transferred into the seminal fluid and cases of AAF due to seminal transfer have been reported, it is now of interest to analyse whether metabolism occurs in the vesicles responsible for the production of seminal fluid and whether this can lead to specific or diagnostic metabolite markers [2,3,4].

Therefore, the use of subcellular fractions in in vitro experiments can be beneficial to gain insights into metabolic activities. In general, liver subcellular fractions are used because xenobiotics are mainly metabolised in the liver by cytochrome P450 (CYP) enzymes and uridine-5′-diphospho-glucuronosyltransferase (UGT) [5]. However, other tissues, such as skin subcellular fractions, can also be used to study the specific metabolism, which in this case is useful for determining subcutaneous and transdermal administration of a drug. To study the (possible) metabolite formation in seminal fluid, obtaining S9 fractions from tissues that contribute significantly to the ejaculate, such as the seminal vesicles or prostate, is a reasonable approach. The seminal vesicle produces a fluid that makes up 50–80% of the ejaculate, and various CYPs have already been detected in the seminal vesicles by real-time polymerase chain reaction (RT-PCR) [6,7,8]. The fluid produced by the seminal vesicle is therefore an important component of the ejaculate and may provide an insight into potential metabolism in the seminal fluid.

The subcellular fraction of the human liver (HL-S9) or seminal vesicle subcellular fraction (SV-S9) are the supernatants obtained from homogenised liver or seminal vesicle tissue after centrifugation at 9000× *g* [9]. This means that cell debris, nuclei, lysosomes and mitochondria are removed, but microsomal and cytosolic fractions remain. The S9 fraction includes the subcellular fraction of the endoplasmic reticulum, which mainly contains CYPs and UGTs. It also includes cytosolic enzymes such as aldehyde oxidase, xanthine oxidase, sulfotransferases, methyltransferases, N-acetyltransferases and glutathione transferases, which may also be important in the metabolism of certain substances [5].

CYPs are a superfamily of haemoproteins responsible for phase-I metabolism, i.e., the oxidative biotransformation of most xenobiotics, with each subfamily having specificity for certain drugs [10,11]. Therefore, the activity of a particular CYP can be determined by analysing the metabolism of model substances. For example, CYP1A2 is known to catalyse the metabolism of phenacetin to acetaminophen, while CYP3A4 promotes the metabolism of midazolam to hydroxy-midazolam [12,13]. In addition to analysing CYP protein activity in the S9 fraction, the cellular expression of CYP enzymes can also be assessed at the transcriptional level, e.g., by using digital PCR (dPCR).

The aim of this study was to perform in vitro experiments with SV-S9 to investigate its potential metabolic activity towards various doping substances. Using typical doping substances such as stanozolol, LGD-4033 and trimetazidine, as well as substances that have been described in connection with potential seminal fluid transfer scenarios (GW1516) and that could frequently be found in seminal fluid (anastrozole), obtaining a first overview of possible metabolite markers was desirable [3,14,15,16]. Further, assessing metabolic contributions of selected and isolated tissues has been considered warranted, especially in comparison to more holistic approaches such as animal experiments reported previously concerning stanozolol, LGD-4033 and their metabolites in seminal fluid [17].

HL-S9 was used as a control to compare the different metabolite profiles with SV-S9 and as an assay quality control. Liquid chromatography coupled to high-resolution/accurate mass spectrometry (LC-HRAM MS) was used to identify both known and novel metabolites. In addition, SV-S9 and HL-S9 were characterised by testing the activity of relevant CYPs by LC-HRAM MS and the cellular expression of CYPs by dPCR.

## 2. Materials and Methods

### 2.1. Chemicals, Kits and Reagents

Acetonitrile (ACN), methanol (MeOH), chloroform and formic acid (FA) were purchased from VWR Chemicals (Langenfeld, Germany). Ultrapure H_2_O was received from Barnstead™ GenPure™ xCAD Plus from Thermo Scientific (Niederelbert, Germany). Nicotinamide adenine dinucleotide phosphate (NADPH reg.) regeneration system was obtained from Promega (Madison, WI, USA). Ethanol (EtOH), potassium dihydrogen phosphate (KH_2_PO_4_), magnesium chloride (MgCl_2_) and reference material of acetaminophen-d_3_, anastrozole, phenacetin, propafenone and stanozolol were obtained from Sigma-Aldrich (St. Louis, MO, USA). Reference material for LGD-4033 was obtained from Selleck-Chem (Houston, TX, USA). High-Capacity cDNA Reverse Transcription Kit, pooled human liver S9 fractions (HL-S9, *n* = 6, 20 mg/mL) and Invitrogen TRIzol Reagent were obtained from Thermo Scientific (Schwerte, Germany). Customised male human seminal vesicle S9 fraction (SV-S9, 0.75 mg/mL, *n* = 1) was obtained from BioIVT (Brussels, Belgium). The QIAcuity Nanoplate 26 k 96-Well and QIAcuity EG PCR Kit were obtained from QIAGEN GmbH (Hilden, Germany). RNA Clean & Concentrator Kit was obtained from Zymo Research Europe GmbH (Freiburg, Germany). Reference material for S-mephenytoin and amodiaquine was obtained from Biomol (Hamburg, Germany), reference material for Trimetazidine was obtained from LGC Standards (Teddington, UK), and reference material for tolbutamide was purchased from Merck (Darmstadt, Germany). The primers used for dPCR were purchased from Biomers (Ulm, Germany) and are listed in Table 1. A total of six CYP enzymes and three housekeeping genes (HG) were tested, which were previously described elsewhere [18,19].

### 2.2. Preparation of Incubation Buffer

For the preparation of the phosphate buffer (100 mM), 3.54 g sodium dihydrogen phosphate (NaH_2_PO_4_) was dissolved in 250 mL of H_2_O (solution A), and 1.75 g of potassium dihydrogen phosphate (KH_2_PO_4_) was dissolved in 125 mL of H_2_O (solution B). Solution B was then added to solution A until a pH of 7.4 was reached (solution C). For the final incubation buffer, 0.25 g MgCl_2_∙6H_2_O were mixed with 250 mL solution C.

### 2.3. In Vitro S9 Fraction Metabolic Assays

A substrate cocktail (CYP-cocktail) for an in vitro assay was prepared as follows: 10,000 µM of tolbutamide and S-mephenytoin, 5000 µM of phenacetin and 500 µM of amodiaquine and propafenone were dissolved in ACN: H_2_O (1:1 (*v*/*v*)). Before use, it was diluted 1:10 (*v*/*v*) in phosphate buffer.

Substances of interest (anastrozole, GW1516, LGD-4033, stanozolol, and trimetazidine) were diluted in 50 mM phosphate buffer (pH 7.4) containing 5 mM MgCl_2_ to produce a stock solution (10 µM each).

To test phase-I metabolism, 10 µL of the stock solution of each substance or CYP-cocktail, 25 µL of NADPH reg. (50 mM) and 6 µL of the HL-S9 fraction (20 mg/mL) or 160 µL of the SV-S9 fraction (0.75 mg/mL) were mixed and made up to a total volume of 200 µL with phosphate buffer to obtain the same protein concentration of 0.6 mg/mL in each sample. Samples were incubated at 37 °C for 24 h. Negative control samples were prepared without S9 fraction and without the substance of interest.

### 2.4. Sample Preparation and Extraction Procedure

Each metabolism sample was quenched by the addition of 600 µL of ice-cold ACN and kept on ice for 20 min. The supernatant was harvested after centrifugation (17,000× g, 10 min) and transferred into a fresh tube. After evaporating using a vacuum centrifuge (70 °C, 90 min), the samples were reconstituted in 100 µL of ACN:H_2_O (20:80 (*v*/*v*)) and diluted before measuring.

### 2.5. LC-HRAM MS Instrumentation and Analytical Conditions

A Vanquish HPLC system, coupled to a Thermo Scientific Orbitrap Exploris 480 (both from Thermo Scientific, Bremen, Germany), was used as the LC-HRAM MS system. Chromatographic separation was achieved using an EC 4/3 Nucleoshell RP 18 Plus guard column (4 × 3 mm, 5 μm particle size; Macherey–Nagel, Düren, Germany) connected to a Poroshell 120 EC-C18 analytical column (50 × 3.0 mm, 2.7 μm particle size; Agilent, Waldbronn, Germany). For each doping substance, an LC-HRAM MS method was developed. Measurements were performed in both positive (anastrozole, GW1516, stanozolol, trimetazidine) and negative ionisation (LGD-4033) modes, with an ionisation voltage of ±3000 V and a transfer tube temperature of 320 °C. For positive mode measurements, H_2_O (A) and ACN (B) with 0.1 % FA each were used as mobile phases at a flow rate of 0.4 mL/min. The gradient for this measurement was as follows: 0–11 min B, starting at 0 % B, increased to 90 %, then increased to 100 %, held for 1 min, then equilibrated at 0 % B for 3 min. For measurements in negative mode the mobile phases were 50 mM ammonium acetate buffer with 0.1 % FA (A) and ACN (B). The gradient was as follows: 0–4.5 min B, starting at 20 %, increased to 50 % B, then increased to 100 % in 2.5 min and held for 1 min. The gradient was then decreased to 70 % B and held for 1.5 min. Then equilibrated at 20 % B for 2 min. The full scan was conducted at a resolution of 60,000 full width at half maximum (FWHM), while MS^2^ experiments (isolation window 1.2 *m*/*z*) were performed at 45,000 FWHM in positive mode and 15,000 FWHM in negative mode. The corresponding ion transitions, normalised collision energies (NCE) and retention times are shown in Table 2. Full scan data were acquired over a *m*/*z* range of 100–800. Nitrogen, supplied by the CMC nitrogen generator (Eschborn, Germany), was used as the collision gas.

A full scan and data-independent acquisition (DIA) modes were set up for the measurements of the protein activity assay. Positive mode with an ionisation voltage of 3500 V and a transfer tube temperature of 350 °C was used. For the full scan, the resolution was set to 45,000 FWHM, and data were acquired over a *m*/*z* range of 100–800. The DIA scan was performed with a resolution of 30,000 FWHM and stepped NCEs of 30 % and 55 %. The isolation window was set at *m*/*z* 100. A total of five DIA experiments were performed: Precursor: *m*/*z* 100–200 from 0 to 14 min, *m*/*z* 200–400 from 5 to 14 min, *m*/*z* 200–400 from 5 to 14 min, *m*/*z* 400–500 from 6 to 14 min, and *m*/*z* 500–600 from 6 to 14 min.

### 2.6. Cell Culture

Undifferentiated HepaRG™ cells (Biopredic International, Rennes, France) were differentiated in-house in William’s E medium containing 10 % foetal bovine serum, 5 µg/mL human insulin, 5 µg/mL gentamicin sulphate, 0.25 µg/mL amphotericin B (all from PAN-Biotech, Aidenbach, Germany), 5 × 10^−5^ M hydrocortisone hemisuccinate (Sigma-Aldrich), 2 mM Glutagrow (Corning, NY, USA) and 2 % DMSO. One million cells were used directly for RNA extraction.

### 2.7. RNA Extraction

Total RNA was extracted from HepaRG™, SV-S9 and HL-S9 fractions using TRIzol/chloroform extraction, and purification was performed using an RNA extraction and purification kit from Zymo Research. For extraction of SV-S9 and HL-S9, 900 µL of TRIzol was mixed with 100 µL of the sample and incubated for 15 min at room temperature (RT). For phase separation, 200 µL of chloroform was added, mixed and incubated for 3 min at RT. The differentiated HepaRG™ cells were incubated with 500 µL of TRIzol, then mixed with 80 µL of chloroform and incubated for 3 min at RT.

The samples were then centrifuged at 12,000× *g* at 4 °C for 15 min. The upper, aqueous phase (RNA) was transferred to a new tube, and an equal volume of ethanol (95–100 %) was added. RNA was purified according to the RNA kit instructions, including the DNAse treatment. In brief, the sample was transferred to the Zymo-Spin IC column in a collection tube and centrifuged for 30 s at 12,000× *g*, RT. For DNAse treatment, 400 µL of wash buffer was added to the column and centrifuged (30 s, 12,000× *g*, RT). The DNAse reaction mix was prepared by mixing 5 µL DNAse I with 35 µL DNAse digestion buffer. This mixture was added directly to the column matrix and incubated at RT for 15 min. Afterwards 400 µL of RNA prep buffer was added to the column and centrifuged (30 s, 12,000× *g*, RT). Then, 700 µL RNA wash buffer was added to the column and centrifuged (30 s, 12,000× *g*, RT). The column was then loaded with 400 µL of RNA Wash Buffer and centrifuged at 12,000× *g* for 1 min to ensure complete removal of the Wash Buffer. For elution of the RNA, 15 µL of RNAse/DNAse-free H_2_O was added to the column matrix and centrifuged (30 s, 12,000× *g*, RT). The quantity and purity of the extracted RNA was tested using a NanoDrop One device (Thermo Fisher Scientific, Waltham, MA, USA). The RNA was stored at −20 °C until further use.

### 2.8. cDNA Reverse Transcription

Reverse transcription was performed using the High Capacity cDNA Reverse Transcription Kit. The master mix was prepared according to the instructions. Briefly, 8 µL of 10× reverse transcription buffer, 3.2 µL of 25× dNTP mix (100 mM), 8 µL of MultiScribe™ reverse transcriptase, and 16.8 µL of nuclease-free H_2_O were prepared on ice. Then 10 µL of the master mix was mixed with 10 µL of RNA sample (HL-S9 113.4 ng/µL (test 1) and 233.6 ng/µL (test 2); SV-S9 153 ng/µL (test 1) and 76.3 ng/µL (test 2); HepaRG™ 200 ng/µL) in a PCR plate and loaded into the thermal cycler (SensoQuest, Göttingen, Germany). The thermal cycler run consisted of 4 steps. The first step was at 25 °C for 10 min, followed by 37 °C for 120 min. The temperature was then increased to 85 °C for 5 min. For the final step, the temperature was cooled to 4 °C and held at this temperature until sample collection. The cDNA was stored at −20 °C until further use.

### 2.9. Digital PCR for Detecting Gene Expression

The dPCR was performed using the QIAcuity EG PCR Kit. In short, the reaction mix was prepared by mixing 4 µL of 3× EvaGreen PCR Master Mix, 3.6 µL of RNase-free H_2_O and 3.2 µL of a primer mix (1.5 µM) containing forward and reverse primers of a CYP or HKG (CYP2B6, CYP2D6, CYP3A4, CYP1A2, CYP2C9, CYP2E1, YHWAZ, UBC, TBP). The reaction mixture containing one primer pair was added to each well of a standard PCR plate. The HL-S9 cDNA was diluted at a 1:5 ratio, and the SV-S9 cDNA was diluted at a 1:2 ratio before adding 1.2 µL in triplicate to the respective wells of the PCR plate. The content of each well from the standard PCR plate was transferred to the wells of the QIAcuity Nanoplate 26 k 8-well (Qiagen, Hilden, Germany). After sealing, the plate was processed and analysed with the QIAcuity One digital PCR system.

The thermal cycling programme consisted of 2 min at 95 °C, 35 cycles of 15 s at 95 °C for denaturation and 15 s at 60 °C for annealing, and a further 15 s at 72 °C for extension. This was followed by cooling at 40 °C for 5 min. Absolute copy number was calculated using Poisson distribution and automatic thresholding using QIAcuity software (V.2.5.0.1 and V.3.1.0.0). A negative control without template (non-template control, NTC, *n* = 3, see supplement) was included to validate the specific PCR reaction for each sample primer set. As a positive control, differentiated HepaRG™ cells (*n* = 3) were tested.

## 3. Results

### 3.1. In Vitro Assay for Doping Substances

The in vitro tests were performed three times (three experiments) with the respective doping substances and measured using targeted LC-HRAM MS methods. Well-established metabolites were identified, and the specific transitions were integrated into LC-HRAM MS methods (see Table 2). The structures of these metabolites can be found in the Supplementary Materials (Figures S6–S10). The data obtained were compared to human (GW1516, anastrozole) or animal (LGD-4033) seminal fluid samples described elsewhere [3,17].

**Figure 1:**
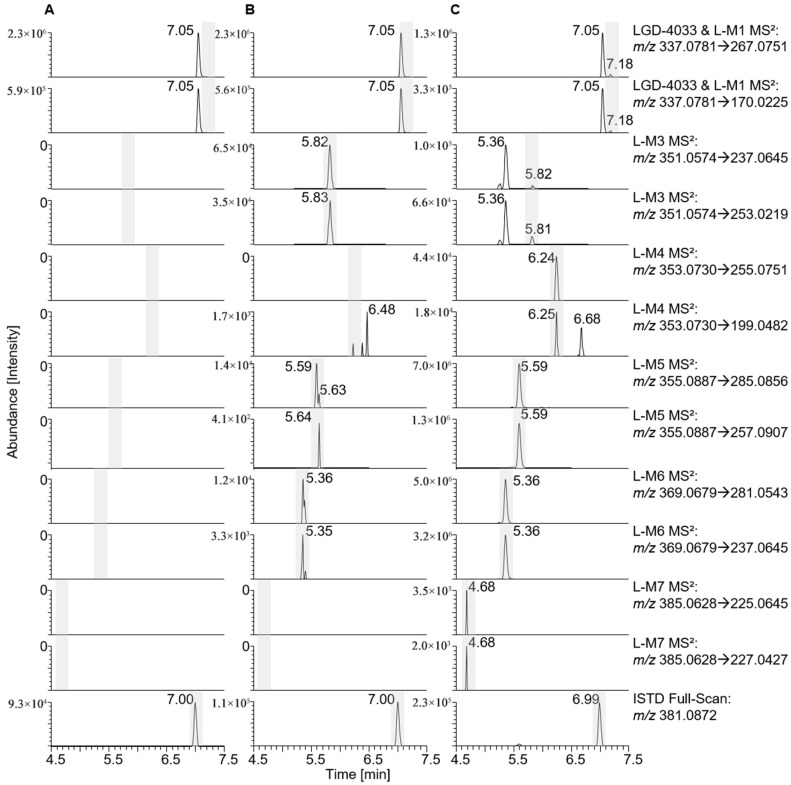
Extracted ion chromatograms of LGD-4033 (**A**) incubated without enzyme, (**B**) incubated with seminal vesicle (SV)-S9, and (**C**) incubated with human liver (HL)-S9, showing the presence of several metabolites in SV-S9 and HL-S9 and the internal standard (ISTD).

The in vitro tests with LGD-4033 yielded several metabolites when incubating the anabolic agent with HL-S9 and SV-S9 fractions (see Table 2, Figure 1 and Figure S6). The hydroxylated metabolite L-M5 was most abundant in the HL-S9 sample. Metabolite L-M3 was the most prominent metabolite in the SV-S9 fraction and showed higher intensities than in the HL-S9 fraction in two out of three experiments. Metabolite L-M5 and metabolite L-M6 were also detected in the SV-S9 fraction in two out of three experiments, but with substantially lower intensities compared to the HL-S9 fraction.

**Figure 2:**
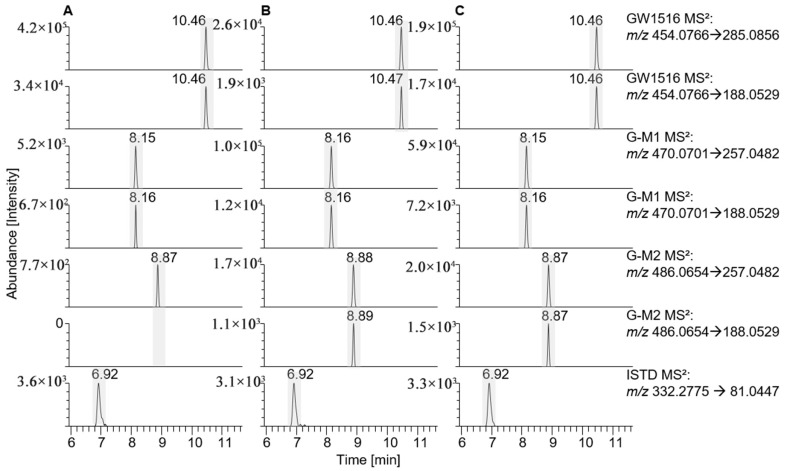
Extracted ion chromatograms of GW1516 (**A**) incubated without enzyme, (**B**) incubated with seminal vesicle (SV)-S9, and (**C**) incubated with human liver (HL)-S9 showing the presence of several metabolites in enzyme blank, SV-S9 and HL-S9 and the internal standard (ISTD).

GW1516 metabolites G-M1-a and G-M2 were observed in samples after incubation with HL-S9 and SV-S9. Small amounts of the metabolite M1-a were present in the enzyme blank, which could be explained by non-enzymatic oxidation, but the intensity was low compared to those in the HL-S9 and SV-S9 fractions (see Figure 2). No M1-b metabolite was found in the SV-S9 and HL-S9 but was detected in vivo [21] (see Table 2).

For trimetazidine, only one metabolite was found in the HL-S9 and SV-S9 fractions, which met the identification criteria applicable to retention time and precursor/product ion pairs (see Table 2). The observed intensities of the metabolite suggest modest conversion when compared to the signal abundance of the intact compound (see Supplemental Figure S1).

No anastrozole metabolites were found in the SV-S9 fraction, and only one anastrozole metabolite was identified at low intensity in HL-S9 (see Table 2, Supplemental Figure S2).

The in vitro experiment showed typical metabolites for stanozolol in the HL-S9 fraction but no metabolites in the SV-S9 fraction (see Table 2). Interestingly, the intensities of the parent substance stanozolol were considerably lower in SV-S9 fractions than in the enzyme blank and in the HL-S9 sample after incubation for 24 h, which could indicate that stanozolol is metabolised in these samples (see Supplemental Figure S3). However, no typical metabolites described in the literature were identified, and also no other metabolites related to stanozolol could be found in positive and negative DIA scans using LC-HRAM MS. Further investigations indicated that stanozolol was bound to proteins and could be extracted from the precipitation pellet, without any metabolites present in this fraction.

### 3.2. In Vitro Protein Activity of CYP Enzymes

**Figure 3:**
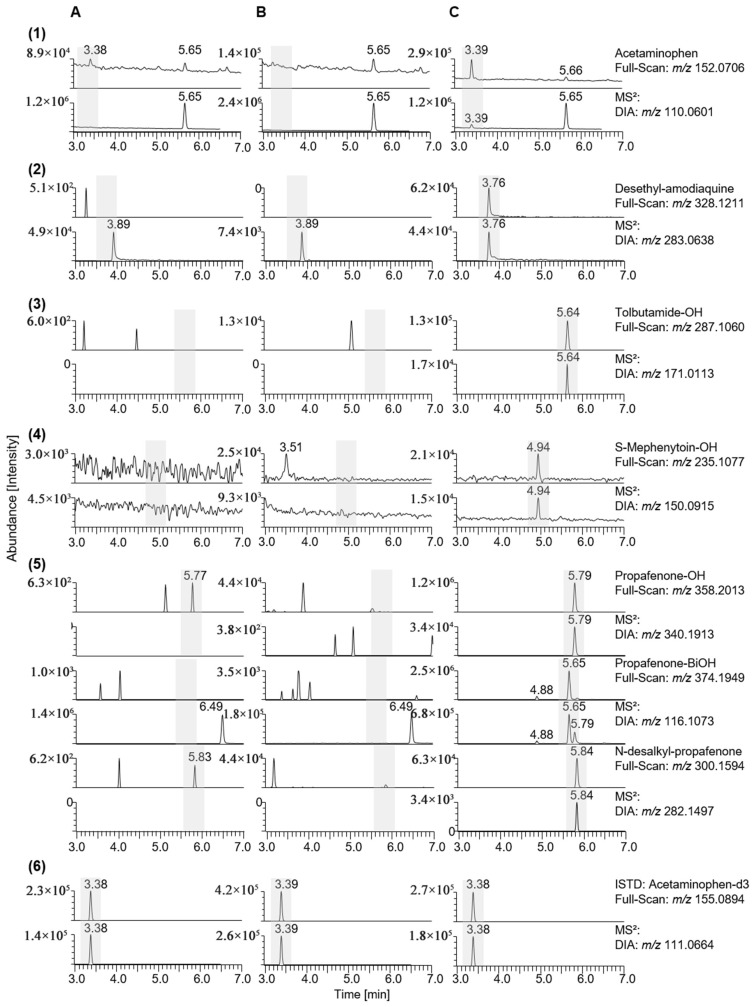
Full-MS-extracted ion chromatograms and MS^2^ extracted ion chromatograms of the three samples incubated with the CYP cocktail (**A**) without enzyme, (**B**) with seminal vesicle (SV)-S9, and (**C**) with human liver (HL)-S9. They show the presence of (**1**) phenacetine, (**2**) amodiaquine, (**3**) tolbutamide, (**4**) S-mephenytoin, and (**5**) propafenone, metabolites only in HL-S9. Part (**6**) shows the spiked internal standard (ISTD) in the three samples.

To elucidate and compare the metabolic capacities of the seminal vesicles and the liver, a protein activity assay was conducted to analyse the characteristic transformation of typical substrates, as already described earlier [19,29]. Therefore, an in vitro test was performed twice to compare the SV-S9 and HL-S9 fractions. In contrast to the protein assay cocktails described previously, midazolam could not be used as a marker for CYP3A4 because the SV-S9 specimen was contaminated with both midazolam and its metabolite [19]. The assay was conducted using LC-HRAM MS in full-scan and DIA mode to detect not only the target metabolites described in the literature [29,30], but also other possible metabolic reactions. The mass spectrometric characteristics of the substances used in the CYP cocktail and their respective metabolites are listed in Table 3. The structures of these metabolites can be found in the Supplementary Materials (Figure S11). Overall, metabolites are formed exclusively in the HL-S9 enzyme-substrate complex (Figure 3C) and no metabolites in the incubation mix without S9 fraction (Figure 3A), indicating that the incubation test is working.

In detail, the CYP1A2 isoform catalyses the metabolic reaction of phenacetin, which leads to the formation of acetaminophen (paracetamol) [31], which could only be found in the HL-S9 incubated sample (see Figure 3((1)C)). No other metabolites of phenacetin were detected. Amodiaquine is metabolised by CYP2C8 to desethyl-amodiaquine [32], which can also only be detected in the sample incubated with HL-S9 (see Figure 3((2)C)). Other metabolites of amodiaquine could not be detected in any incubation. CYP2C9 catalyses the hydroxylation of tolbutamide [33], which was detected in the sample incubated with HL-S9, and no further metabolites could be detected (see Figure 3((3)C)). S-Mephenytoin is metabolised by CYP2C19 to form hydroxy-mephenytoin [34], which can only be observed in the sample incubated with HL-S9 (see Figure 3((3)C)). No further metabolites were found here either. Propafenone is metabolised by CYP2D6 to hydroxy-propafenone, as described in the literature [35], but the HL-S9 sample shows a metabolite with *m*/*z* 374.1949 in addition to the hydroxy metabolite, which could indicate twofold-hydroxylation. This metabolite is clearly more intense in the chromatogram than the hydroxy metabolite, and they share the production *m*/*z* 116.1069 (see Figure 3((5)C)). Propafenone is also dealkylated by CYP3A4 and CYP1A2 [35], which can be observed in the sample incubated with HL-S9.

### 3.3. Gene Expression of Different CYPs

The dPCR was used to probe for gene expressions of CYPs in HL-S9 and SV-S9, performed twice at different time points. While two different HL-S9 donor pools existed, which may explain the observed deviations in RNA concentration and gene expression between the different experiments for the HL-S9 fractions, material from only one donor was available for the SV-S9. In the first experiment (test 1), RNA concentrations of 226.8 ng/µL HL-S9 and 15.3 ng/µL SV-S9 were quantified, while in the second experiment (test 2), concentrations of 1168.25 ng/µL HL-S9 and 7.63 ng/µL SV-S9 were measured. Purity determined by A260/A280 and A260/A230 ratios gave acceptable values for the HL-S9 sample (test 1: 2.00/1.91; test 2: 2.06/2.07) and lower values for the SV-S9 sample (test 1: 1.78/1.65; test 2: 1.65/0.92), attributable to some degree of contamination. For example, increased absorbance at 230 nm in RNA samples may be due to contamination by guanidine thiocyanate, which absorbs strongly at 220–230 nm and is present at very high concentrations in the extraction reagent (TRIzol) [36]. Further clean-up of the SV-S9 RNA did not improve the A260/A230 but resulted in loss of RNA and was therefore not pursued.

**Figure 4:**
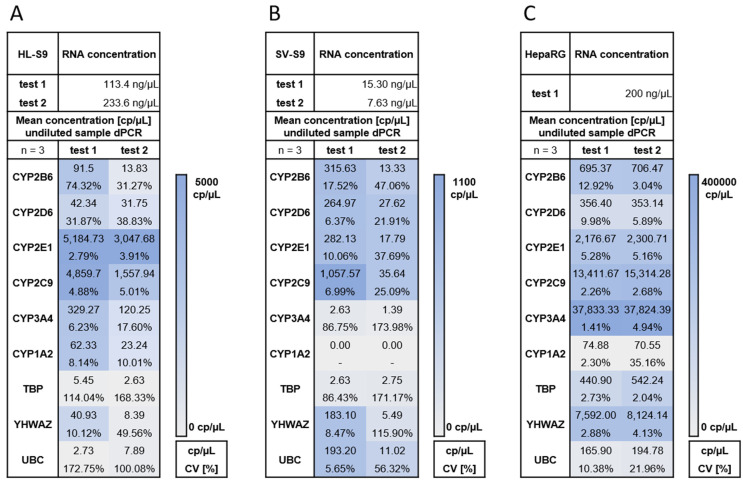
RNA concentration and mean concentration of undiluted sample [cp/µL] of CYPs and housekeeping genes (TBP, YHWAZ, UBC) in two experiments (test 1, test 2) in (**A**) human liver (HL)-S9, (**B**) seminal vesicle SV-S9, and (**C**) positive control (HepaRG). The blue–white–grey spectrum indicates the high/low/no expression level for each gene; The dPCR samples were measured in triplicate (*n* = 3) and the coefficient of variation (CV) is indicated below the sample.

The dPCR experiments were performed with primers for the detection of CYP2B6, CYP2D6, CYP3A4, CYP1A2, CYP2C9, CYP2E1 and the HKGs UBC, TBP and YHWAZ (see Table 1), which have been used for quantitative PCR before [18,19]. Due to the very limited information about the CYP expression in the human seminal vesicle [8], HKGs were included as additional dPCR controls for the SV-S9 fraction but were not used as a normalisation variable for quantification. Here, the low RNA concentration and potential contamination would not allow for a reliable quantification. The presence of residual genomic DNA by direct addition of a portion of the isolated RNA to the dPCR was not tested due to the limited sample volume of the SV-S9 fraction. Therefore, it cannot be excluded that genomic DNA could bias the results, but thorough DNAse treatment was performed during RNA purification to ensure that no genomic DNA was present in the sample. After an initial dPCR test with the CYP2B6 primer pair, the concentration of HL-S9 was increased and the experiment repeated (see Supplemental Figures S6 and S7). All subsequent dPCR measurements were then performed with the adjusted concentration as described in the methods section. The calculated mean concentration of undiluted samples of CYPs in HL-S9, SV-S9 and HepaRG™ in two experiments (test 1, test 2) are shown in Figure 4. Due to the different RNA input concentrations used for HL-S9, SV-S9 and HepaRG™, the expression values [cp/µL] cannot be compared. However, both experiments show that CYP2B6, CYP2D6, CYP2E1, and CYP2D9 are expressed in SV-S9. CYP2B6, CYP2D6, CYP2E1, CYP2C9, CYP3A4, and CYP1A2 were found in HL-S9. The SV-S9 and HL-S9 were also tested for HKGs, showing low values in HL-S9 and SV-S9, except for UBC and YHWAZ in SV-S9 in the first test (see Figure 4). Detailed information on scatter plots, fluorescence intensity and thresholds for all experiments can be found in the Supplementary Materials.

## 4. Discussion

### 4.1. In Vitro Assay for Doping Substances

The protein concentration (0.6 mg/mL) of the HL-S9 fraction used in this study was adjusted to the concentration of the SV-S9 fraction in order to operate the assays under comparable conditions. As these protein concentrations were lower than those employed in earlier studies [37], the herein presented results of the HL-S9 incubation experiments are not immediately comparable to other in vitro studies.

The metabolism of LGD-4033 has been studied in great detail, and the occurrence of this substance in doping control samples has increased in recent years, possibly due to highly sensitive analytical test methods and/or contamination scenarios [15]. While it has been demonstrated that LGD-4033 and its metabolites can occur in seminal fluid, it remains to be clarified whether they are formed in the seminal vesicle [17]. Since no metabolites were detected in the enzyme blank, the presence of three metabolites identified in two out of three SV-S9 experiments suggests some metabolic activity of the SV-S9 fraction. Analytical results obtained from samples prepared after incubation of LGD-4033 in SV-S9 or HL-S9 and from an in vivo experiment, described previously, differ in their patterns of main metabolites, indicating different enzymatic activities and processes [17]. Of note, no metabolite exclusive to SV-S9 was observed.

Stanozolol has been widely abused in sports until today, although modern mass spectrometry techniques allow for highly sensitive detection of stanozolol and its metabolites. In addition, it is a good model substance because of its well-studied metabolism in the anti-doping context [14,26,38,39]. Several metabolites were found in the HL-S9 fraction, indicating the principal functionality of the assay, but no metabolites of stanozolol were found in SV-S9.

In a previous study, a number of human seminal fluid samples were found to contain anastrozole and its metabolites [3]. This suggests that anastrozole may be a potential contaminant in urine samples of female athletes after unprotected intimate contact. However, only one phase-I metabolite of anastrozole could be detected in the HL-S9 fraction and none in the SV-S9 fraction. As the intensity of anastrozole in SV-S9 is similar to the enzyme blank, no extensive metabolism is expected. Therefore, these tests could not provide any further evidence/markers for the seminal fluid contamination scenario.

GW1516 has also been detected in a seminal fluid sample in a previous study, in which a case scenario is described where a professional athlete tested positive for GW1516 metabolites and denied intentional use. The athlete’s partner, who confirmed the use of GW1516, was identified as a possible source of exposure through intimate contact [2,4]. As metabolites of GW1516 were found in the SV-S9 fraction (Figure 2), it is possible that GW1516 is further metabolised in the seminal vesicles.

### 4.2. In Vitro Protein Activity of Different CYPs

The absence of metabolites typically formed by common CYPs in the SV-S9-incubated samples indicated very low concentrations or no presence of the investigated CYPs, translating to very low or no metabolic activity in the seminal vesicle, at least concerning the assessed typical/frequent hepatic CYPs.

### 4.3. Gene Expression of Different CYPs

A limitation of this experiment, apart from the availability of only one SV-S9 donor, was the very low RNA concentration of the SV-S9 sample. This negatively affected the number of positive partitions in the dPCR, which had to be considered when comparing the experiments (see Supplemental Figures S6–S16), where the same CYPs were found in both SV-S9 experiments, although differing in the observed concentrations.

CYP2D6 expression was detected in the SV-S9 fraction, although no activity was observed on a protein level in the metabolism test. CYP2D6 contributes to the metabolisation of many prescribed drugs and has been detected in various tissues such as the liver, brain, intestinal tissue, lymphoid cells and also seminal vesicles [8,40,41,42]. But CYP2D6 is also a highly polymorphic gene, and variations in the CYP genes can influence the expression and function of the enzymes [10,40,41,43]. For example, one study showed that minor changes in the structure of the active site of CYP2D6, such as the substitution of a single amino acid residue (F483) in CYP2D6, are sufficient to convert the enzyme to a steroidal 15α-hydroxylase, thus presenting a function that had not previously prevailed and thereby affecting the substrate specificity of the enzyme [44,45]. As the samples were not genotyped, it cannot be excluded that CYP2D6 has a different function and expression level in the SV-S9 fraction than in the HL-S9 fraction. In addition, SV-S9 is from a single donor and HL-S9 is from pooled donors (*n* = 6), which also influences the results with regard to the described polymorphic character of CYP2D6.

CYP2E1 is mainly found in the liver and plays an important role in metabolising potential toxic substrates (such as ethanol) to even more toxic products [46,47]. CYP2E1 was detected in HL-S9 and also in SV-S9. CYP2E1 has previously been reported to be present in the seminal vesicle and rat testis, but not in the prostate [8,45,48]. For some CYPs, such as CYP2E1, it is also important to note that mRNA levels do not necessarily correlate with enzyme activity, as studies have shown pre-transcriptional, pre-translational, translational and post-translational regulation of CYP2E1 expression [10,49,50]. In fact, one study showed high mRNA levels of CYP2E1 (most abundant together with CYP3A4), but no correlation with protein activity [43]. Therefore, in this case, a high gene expression level of this CYP enzyme does not necessarily correspond to a metabolic activity.

CYP2C9 is one of the most abundantly expressed CYP2C isoforms and the third most important CYP in the liver after CYP3A4 and CYP2D6 [51,52]. The gene expression detected by dPCR in all samples is relatively high. This is the first time that CYP2C9 has been detected in the seminal vesicles, which is in contrast to a previous study [8]. CYP2C9 was found previously in the liver, kidney, testis, adrenal gland, ovary and duodenum [53]. The lack of CYP2C9 protein activity in seminal vesicles despite the presence of mRNA can be explained by a study showing no correlation between mRNA levels and protein activity, with polymorphic variants also possibly contributing to lower activity [43].

CYP3A4 is the major human enzyme for the metabolism of xenobiotics and the most abundantly expressed CYP in the human liver [54,55]. Midazolam, the commonly used model substance, could not be used because impurities of midazolam and hydroxy-midazolam were found in the SV-S9. CYP3A4 activity was only observed in HL-S9 in this study, corroborated by the formation of the metabolite N-desalkyl-propafenone [35,56]. No metabolites of propafenone were detected in SV-S9. The SV-S9 manufacturer’s CYP3A4 assay (using midazolam and testosterone) also showed no metabolic activity. Consistent with this, no CYP3A4 mRNA expression was detected in SV-S9.

The CYP1A subfamily is one of the most abundant CYP families and consists of two members, 1A1 and 1A2, which are mainly found in the human liver [12,57]. Therefore, it is plausible that CYP1A2 could not be found in SV-S9, as no protein activity and no signal in dPCR were found. In contrast, previous studies reported on CYP1A2 mRNA in the seminal vesicle and in the prostate [8,45]. As CYP1A2 was only present in small quantities in the positive control (HepaRG™) in this study, it is, of course, possible that the quantities in SV-S9 are below the detection limit.

To normalise raw values obtained from qPCR, housekeeping genes (HKG) are commonly used as internal standards [58]. Since dPCR can be used to measure absolute transcript levels, results do not need to be expressed relative to HKGs, but for quantification it is recommended to use HKGs for normalisation [59]. In this qualitative study, however, probing for the presence of HKGs such as UBC, TBP and YWHAZ in the S9 fractions and verifying the PCR assay’s functionality was considered relevant.

UBC was detected in SV-S9 but not in HL-S9. This is consistent with the literature, as ubiquitin is present in human seminal plasma and plays an important role in regulating sperm quality and male fertility [60,61].

TBP was not detected in SV-S9 and HL-S9. TBP is a suitable HKG for qPCR and is recommended for normalisation because of the high stability of the gene [58,62]. However, it also shows a relatively low expression level compared to other HKGs such as glyceraldehyde-3-phosphate dehydrogenase (GAPDH), beta-actin (ACTB), and 18S ribosomal RNA (18S) [58,62].

The chaperone protein YWHAZ has also previously been identified as a suitable reference gene [58,63] and was detected in HL-S9 and SV-S9 in low levels, which is in line with the literature [58], so that overall the functionality of the employed dPCR was confirmed.

## 5. Conclusions

Using in vitro metabolism assays and gene expression analysis, this study provides a comprehensive assessment of the metabolic and transcriptional activity of the seminal vesicle in comparison to the liver, with a particular focus on the metabolism of doping agents. The results indicate that, although certain metabolic transformations occur in the SV-S9 fraction, the overall metabolic potential remains substantially lower than that of the HL-S9 fraction. Modest metabolism of LGD-4033, GW1516 and trimetazidine was observed in SV-S9, while no metabolic transformation of stanozolol or anastrozole was detected.

Metabolic markers exclusive to seminal vesicle metabolism would offer distinct target analytes to identify ejaculate-borne contaminations in female athletes’ doping control urine samples, supporting result interpretation and decision-making processes in cases of AAFs. The obtained data suggest that the metabolic contributions from seminal vesicles are minimal and no specific biotransformation products were identified. However, other techniques have been proposed to provide analytical insight into this specific contamination scenario, such as monitoring specific seminal fluid proteins in the athlete’s urine or complementary hair testing [4,64]. Hair testing in particular has led to successful outcomes for athletes in tribunals in recent years and could be considered as a method of confirming unintentional ingestion [65].

A major limitation of this study was that SV-S9 was available from a single donor only. Future studies investigating inter-individual variability, potential unidentified enzymatic pathways and the effect of genetic polymorphisms on CYP expression in the seminal vesicle appear warranted. Furthermore, our approach only identifies metabolites that can be detected in vitro using SV-S9. As previous studies have shown that in vitro and in vivo systems can produce diverging results, it is important to highlight the limitations of using only an in vitro model and emphasise the need for further research [66,67].

The presented findings are consistent with previous studies and confirm that the seminal vesicle plays a negligible role in the metabolism of xenobiotics, including doping agents. Importantly, with the lack of unique metabolic markers in seminal fluid, the differentiation between contamination and intentional drug administration in anti-doping investigations remains challenging.

## Figures and Tables

**Table 1:** Forward (for) and reverse (rev) primers used for dPCR.

Primer	Sequence (Forward)	Sequence (Reverse)
CYP1A2	5′-ctc ctc ctt ctt gcc ctt ca-3′	5′-gta gaa gcc att cag cgt tgt g-3′
CYP2B6	5′-ttc cta ctg ctt ccg tct atc aaa-3′	5′-gtg cag aat ccc aca gct ca-3′
CYP2C9	5′-aag gag atc cgg cgt ttc tc-3′	5′-cgg tcc tca atg ctc ctc ttc-3′
CYP2D6	5′-gac cag aga tgg gtg acc ag-3′	5′-cga tgt cac ggg atg tca ta-3′
CYP2E1	5′-cat gag att cag cgg ttc atc-3′	5′-ggt gtc tcg ggt tgc ttc a-3′
CYP3A4	5′-tca gcc tgg tgc tcc tct atc tat-3′	5′-aag ccc tta tgg tag gac aaa ata ttt-3′
TBP	5′-gag agt tct ggg att gta ccg-3′	5′-atc ctc atg att acc gca gc-3′
UBC	5′-gcc tta gaa ccc cag tat cag-3′	5′-aag aaa acc agt gcc cta gag-3′
YHWAZ	5′-atg caa cca aca cat cct atc-3′	5′-gca tta tta gcg tgc tgt ctt-3′

**Table 2:** Mass spectrometric characteristics of anastrozole, GW1516, LGD-4033, stanozolol, and trimetazidine.

Analyte	Exact Mass [*m*/*z*]	Formula	Retention Time [min]	NCE [%]	Product Ions [*m*/*z*]	Formation In Vitro/In Vivo	Literature
Anastrozole	294.1713	C_17_H_19_N_5_	7.13	30	225.1386		
					266.1652		
A-M3	310.1662	C_17_H_19_N_5_O	5.84	30	241.1335	HL-S9	[3,20]
					214.1226		
GW1516	454.0766	C_21_H_19_O_3_NF_3_S_2_	10.44	50	257.0482		
					188.0529		
G-M1-a	470.0701	C_21_H_19_O_4_NF_3_S_2_	8.10	50	274.0508	HL-S9, SV-S9, in vivo	[21,22]
					257.0482		
G-M1-b			8.98	50	274.0508	in vivo	[21]
					257.0482		
G-M2	486.0654	C_21_H_19_O_5_NF_3_S_2_	8.83	50	272.0346	HL-S9, SV-S9, in vivo	[21,22]
					257.0482		
LGD-4033	337.0781	C_14_H_11_F_6_N_2_O^-^	7.05	30	267.0751		
					170.0225		
L-M1			7.18		267.0751	HL-S9, in vivo	[15,23,24]
					239.0438		
L-M3	351.0574	C_14_H_9_F_6_N_2_O_2_^-^	5.82	30	237.0645	SV-S9, HL-S9, in vivo	[15,24,25]
					253.0219		
L-M4	353.073	C_14_H_11_F_6_N_2_O_2_^-^	6.24	30	255.0751	HL-S9, in vivo	[15,23,24]
					199.0492		
L-M5	355.0887	C_14_H_13_F_6_N_2_O_2_^-^	5.59	30	285.0856	HL-S9, SV-S9, in vivo	[15,23,24]
					257.0907		
L-M6	369.0679	C_14_H_11_F_6_N_2_O_3_^-^	5.36	30	281.0543	HL-S9, SV-S9, in vivo	[15,23,24,25]
					170.0212		
L-M7	385.0628	C_14_H_11_F_6_N_2_O_4_^-^	4.68	30	225.0645	HL-S9	[15,23]
					227.0427		
Stanozolol	329.2598	C_21_H_33_N_2_O^+^	6.92	65	81.0447	-	
					107.0855		
S-M3-a	345.2537	C_21_H_33_N_2_O_2_^+^	4.9	65	81.0447	HL-S9	[14,26,27]
					95.0855		
S-M3-b			5.01		81.0447	HL-S9	
					95.0604		
S-M3-c			5.58		81.0447	HL-S9	
					95.0604		
S-M3-d			5.74		81.0447	HL-S9	
					95.0604		
S-M3-e			6.08		97.0396	HL-S9	
					121.1012		
S-M3-f			6.27		145.076	HL-S9	[14,26]
					95.0855		
S-M4-a	361.2486	C_21_H_33_N_2_O_3_^+^	4.4	65	81.0447	HL-S9	[14,27]
					95.0604		
S-M4-b			4.52		81.0447	HL-S9	
					361.2486		
S-M4-c			4.89		81.0447	HL-S9	
					361.2486		
S-M4-d			5.05		97.0396	HL-S9	
					95.0855		
S-M4-e			5.36		145.076	HL-S9	[14]
					95.0604		
S-M5	343.238	C_21_H_31_N_2_O_2_^+^	5.68	65	81.0447	HL-S9	[26,27]
					257.2012		
S-M6	359.2329	C_21_H_31_N_2_O_3_^+^	5.05	65	97.0396	HL-S9	[26,27]
					273.1961		
Trimetazidine	267.1703	C_14_H_22_N_2_O_3_	5.06	30	181.0858		
					166.0629		
T-M1	283.1649	C_14_H_23_N_2_O_4_	4.50	30	181.0858	HL-S9, SV-S9	[16,28]
					166.0629		
Paroxetine-d_6_	336.1876		6.67	45	76.0995		
S-24	381.0868	C_18_H_13_O_3_N_2_F_4_^-^	7.00	30	241.0594		
Stanozolol-d_3_	332.2275		6.97	65	81.047		

**Table 3:** Mass spectrometric characteristics of the substances used in the CYP cocktail (bold) and their respective metabolites.

Analyte	Formula	Retention Time [min]		[*m*/*z*]	Formation In Vitro
Phenacetin	C_10_H_13_NO	5.65	Full-Scan	180.1019	-
			MS^2^	138.0915	
Acetaminophen (CYP1A2)	C_8_H_9_NO_2_	3.39	Full-Scan	152.0706	HL-S9
			MS^2^	110.0601	
Amodiaquine	C_20_H_22_ClN_3_O	3.89	Full-Scan	356.1524	-
			MS^2^	283.0638	
desethyl-amodiaquine (CYP2C8)	C_18_ClN_3_O	3.76	Full-Scan	328.1211	HL-S9
			MS^2^	283.0638	
S-mephenytoin	C_12_H_14_N_2_O_2_	6.17	Full-Scan	219.1128	-
			MS^2^	134.0967	
OH-mephenytoin (CYP2C19)	C_12_H_14_N_2_O_3_	4.94	Full-Scan	235.1077	HL-S9
			MS^2^	150.0915	
Tolbutamide	C_12_H_20_N_2_O_3_S	7.44	Full-Scan	271.1110	-
			MS^2^	155.0165	
OH-tolbutamide (CYP2C9)	C_12_H_20_N_2_O_4_S	5.64	Full-Scan	287.1060	HL-S9
			MS^2^	171.0113	
Propafenone	C_21_H_27_NO_3_	6.49	Full-Scan	342.2063	-
			MS^2^	116.1073	
OH-propafenone (CYP2D6)	C_21_H_27_NO_4_	5.79	Full-Scan	358.2013	HL-S9
			MS^2^	340.1913	
Bi-OH-propafenone	C_21_H_27_NO_5_	5.65	Full-Scan	374.1949	HL-S9
			MS^2^	116.1069	
N-desalkyl-propafenone (CYP3A4/CYP1A2)	C_18_H_22_NO_3_	5.82	Full-Scan	300.1594	HL-S9
			MS^2^	282.1497	
Acetaminophen-d_3_	-	3.38	Full-Scan	155.0894	-
			MS^2^	111.0664	

## Data Availability

Data is contained within the article or Supplementary Materials.

## Supplementary Materials

The following supporting information can be downloaded at: https://www.mdpi.com/article/10.3390/metabo15070452/s1, Figure S1: Extracted ion chromatograms of trimetazidine (top test 1, middle test 2, bottom test 3) (A) incubated without enzyme, (B) incubated with SV-S9 and (C) with HL-S9 showing the presence of one metabolite in SV-S9 and HL-S9. Figure S2: Extracted ion chromatograms of anastrozole (top test 1, middle test 2, bottom test 3) (A) incubated without enzyme, (B) incubated with SV-S9 and (C) with HL-S9 showing the presence of one metabolite only in HL-S9. Figure S3: Extracted ion chromatograms of stanozolol (top test 2, bottom test 3) (A) incubated without enzyme, (B) incubated with SV-S9 and (C) with HL-S9 showing the presence of several metabolites only in HL-S9. Figure S4: Extracted ion chromatograms of GW1516 (top: test 1, bottom test: 2) (A) incubated without enzyme, (B) incubated with SV-S9 and (C) with HL-S9 showing the presence of several metabolites only in HL-S9. Figure S5: Extracted ion chromatograms of LGD-4033 (top: test 1, bottom test: 2) (A) incubated without enzyme, (B) with SV-S9 and (C) with HL-S9 showing the presence of several metabolites only in HL-S9. Figure S6: Structure of LGD-4033 and the tentative identified metabolites in vitro using HL-S9 (shown in blue) and SV-S9 (shown in grey) are displayed. Figure S7: Structure of GW1516 and the tentative identified metabolites in vitro using HL-S9 (shown in blue) and SV-S9 (shown in grey) are displayed. Figure S8: Structure of anastrozole and the tentative identified metabolite in vitro using HL-S9 (shown in blue) are displayed. Figure S9: Structure of trimetazidine and the tentative identified metabolite in vitro using HL-S9 (shown in blue) and SV-S9 (shown in grey) are displayed. Figure S10: Structure of stanozolol and the tentative identified metabolites in vitro using HL-S9 (shown in blue) are displayed. Figure S11: Structures of CYP substrates and the tentative identified metabolites in vitro using HL-S9 (shown in blue) are displayed. Figure S12: 1-D scatter plot (test 1) of HL-S9, SV-S9, NTC and PC samples for dPCR using CYP2B6 primers. Figure S13: 1-D scatter plot (test 1) of a repeat measurement of HL-S9 samples with increased concentration compared to the previous measurement for dPCR using CYP2B6 primers. The adjusted concentration of HL-S9 was used for further measurements. Figure S14: 1-D scatter plot (test 2) of HL-S9, SV-S9, NTC and PC samples for dPCR using CYP2B6 primers. Figure S15: 1-D scatter plots (test 1, test 2) of HL-S9, SV-S9, NTC and PC samples for dPCR using CYP2D6 primers. Figure S16: 1-D scatter plots (test 1, test 2) of HL-S9, SV-S9, NTC and PC samples for dPCR using CYP2C9 primers. Figure S17: 1-D scatter plots (test 1, test 2) of HL-S9, SV-S9, NTC and PC samples for dPCR using CYP1A2 primers. Figure S18: 1-D scatter plots (test 1, test 2) of HL-S9, SV-S9, NTC and PC samples for dPCR using CYP2E1 primers. Figure S19: 1-D scatter plots (test 1, test 2) of HL-S9, SV-S9, NTC and PC samples for dPCR using CYP3A4 primers. Figure S20: 1-D scatter plots (test 1, test 2) of HL-S9, SV-S9, NTC and PC samples for dPCR using TBP primers. Figure S21: 1-D scatter plots (test 1, test 2) of HL-S9, SV-S9, NTC and PC samples for dPCR using UBC primers. Figure S22: 1-D scatter plots (test 1, test 2) of HL-S9, SV-S9, NTC and PC samples for dPCR using YWHAZ primers.

## Abbreviations

The following abbreviations are used in this manuscript:

AAF	Adverse analytical finding
LC	Liquid chromatography
HRAM MS	High-resolution/accurate mass spectrometry
dPCR	digital polymerase chain reaction
