# Supplementary material for: In Vitro Metabolism of Doping Agents (Stanozolol, LGD-4033, Anastrozole, GW1516, Trimetazidine) by Human Seminal Vesicle and Liver Fractions
Source: Metabolites. 2025 Jul 4;15(7):452. doi: 10.3390/metabo15070452 (PMC12298852; doi:10.3390/metabo15070452)
Supplement: Supplementary file 1 [file metabolites-15-00452-s001.zip › metabolites-3726403-supplementary.pdf]

SUPPLEMENTAL INFORMATION:

Title: In vitro metabolism of doping agents (stanozolol, LGD-4033, anastrozole, GW1516, trimetazidine) by seminal vesicle and pooled human liver fractions

Author information

J Sternberg[1] (ORCID: 0009-0007-8423-8604), I Peters[1], N Naumann[1] (ORCID: 0009-0006-1467-442X), A Thomas[1] (ORCID: 0000-0003-1199-0743), M Thevis[1,2] (ORCID: 0000-0001-5972-8854)

[1] Institute of Biochemistry, Center for Preventive Doping Research, German Sport University Cologne, Cologne, Germany

[2] European Monitoring Center for Emerging Doping Agents (EuMoCEDA), Cologne/Bonn, Germany

Corresponding author:

Mario Thevis, PhD, Center for Preventive Doping Research – Institute of Biochemistry,

Email: thevis@dshs-koeln.de

The supplementary material includes the following data:

1. **Extracted ion chromatograms of sample incubated with *trimetazidine***
2. **Extracted ion chromatograms of sample incubated with *anastrozole***
3. **Extracted ion chromatograms of sample incubated with *stanozolol***
4. **Extracted ion chromatograms of sample incubated with *LGD-4033***
5. **Extracted ion chromatograms of sample incubated with *GW1516***
6. **Structures of *LGD-4033* and tentatively identified metabolites *in vitro***
7. **Structures of *GW1516* and tentatively identified metabolites *in vitro***
8. **Structures of *anastrozole* and tentatively identified metabolites *in vitro***
9. **Structures of *trimetazidine* and tentatively identified metabolites *in vitro***
10. **Structures of *stanozolol* and tentatively identified metabolites *in vitro***
11. **Structures of *CYP-substrates* and tentatively identified metabolites *in vitro***
12. **Supplemental: Scatter plots of dPCRs of different CYP enzyme genes**
13. **Supplemental: Scatter plots of dPCRs of different housekeeping genes**

1. Extracted ion chromatograms of sample incubated with *trimetazidine*

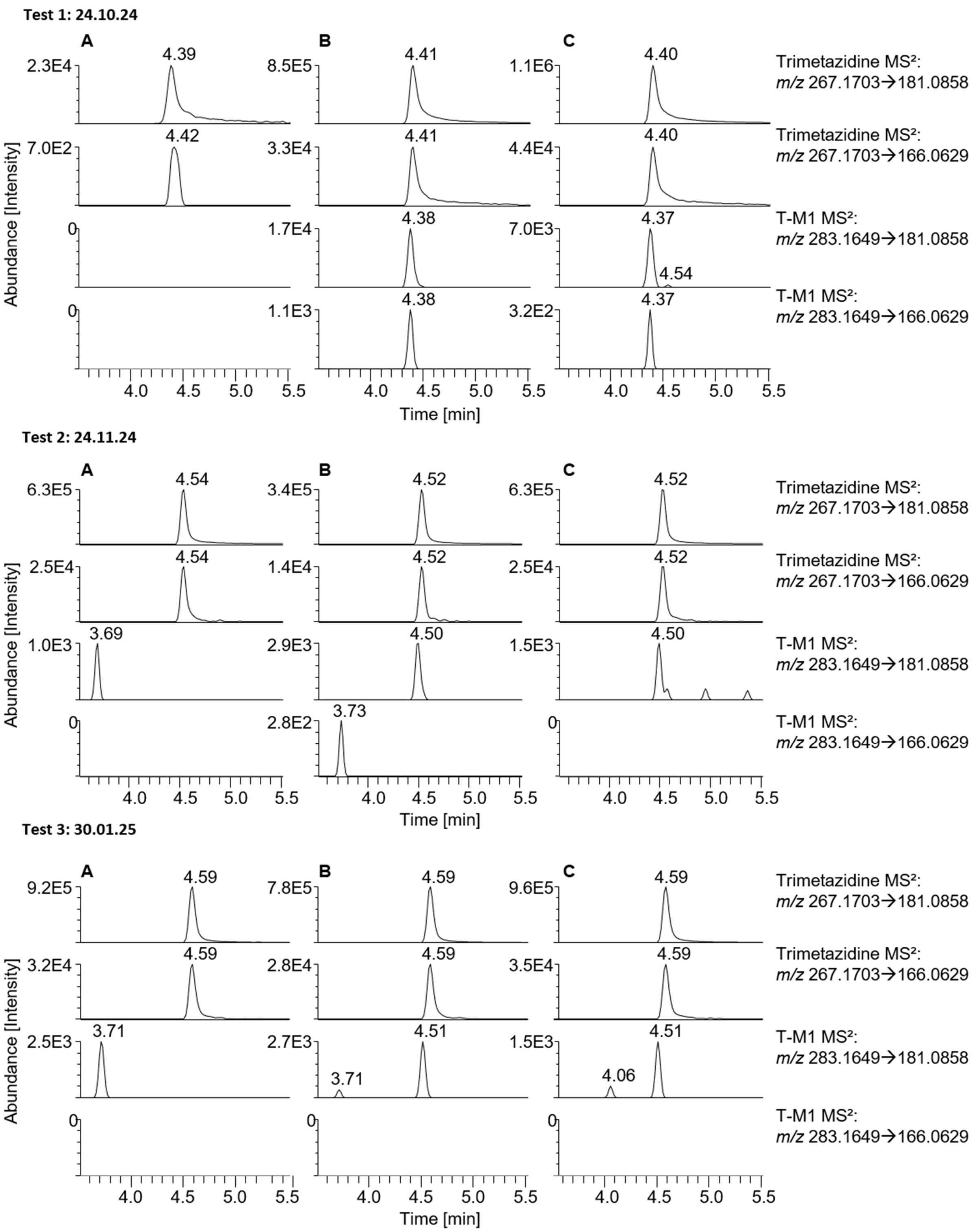

Figure S1: Extracted ion chromatograms of trimetazidine (top test 1, middle test 2, bottom test 3) (A) incubated without enzyme, (B) incubated with SV-S9 and (C) with HL-S9 showing the presence of one metabolite in SV-S9 and HL-S9.

2. Extracted ion chromatograms of sample incubated with *anastrozole*

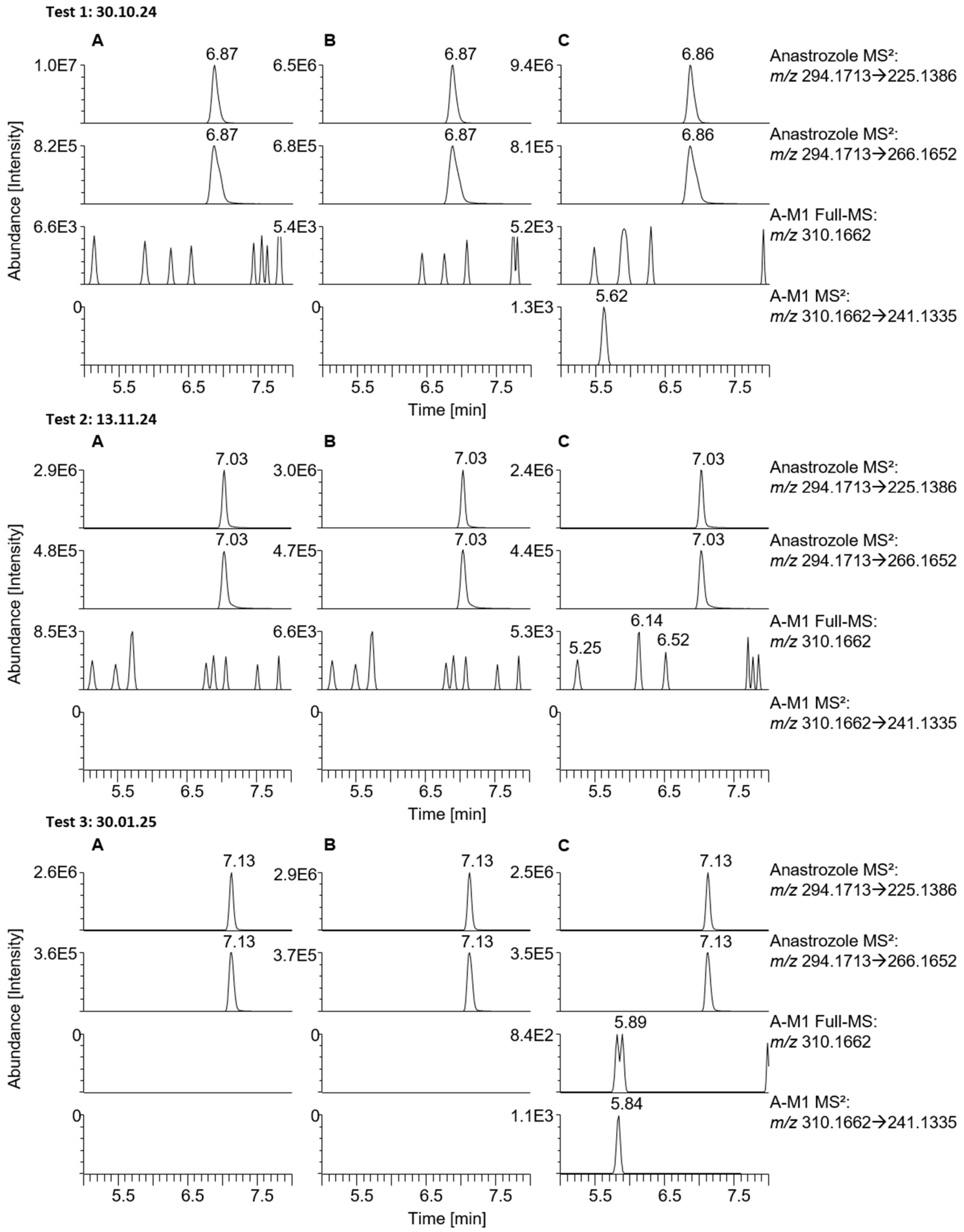

Figure S2: Extracted ion chromatograms of anastrozole (top test 1, middle test 2, bottom test 3) (A) incubated without enzyme, (B) incubated with SV-S9 and (C) with HL-S9 showing the presence of one metabolite only in HL-S9.

3. Extracted ion chromatograms of sample incubated with *stanozolol*

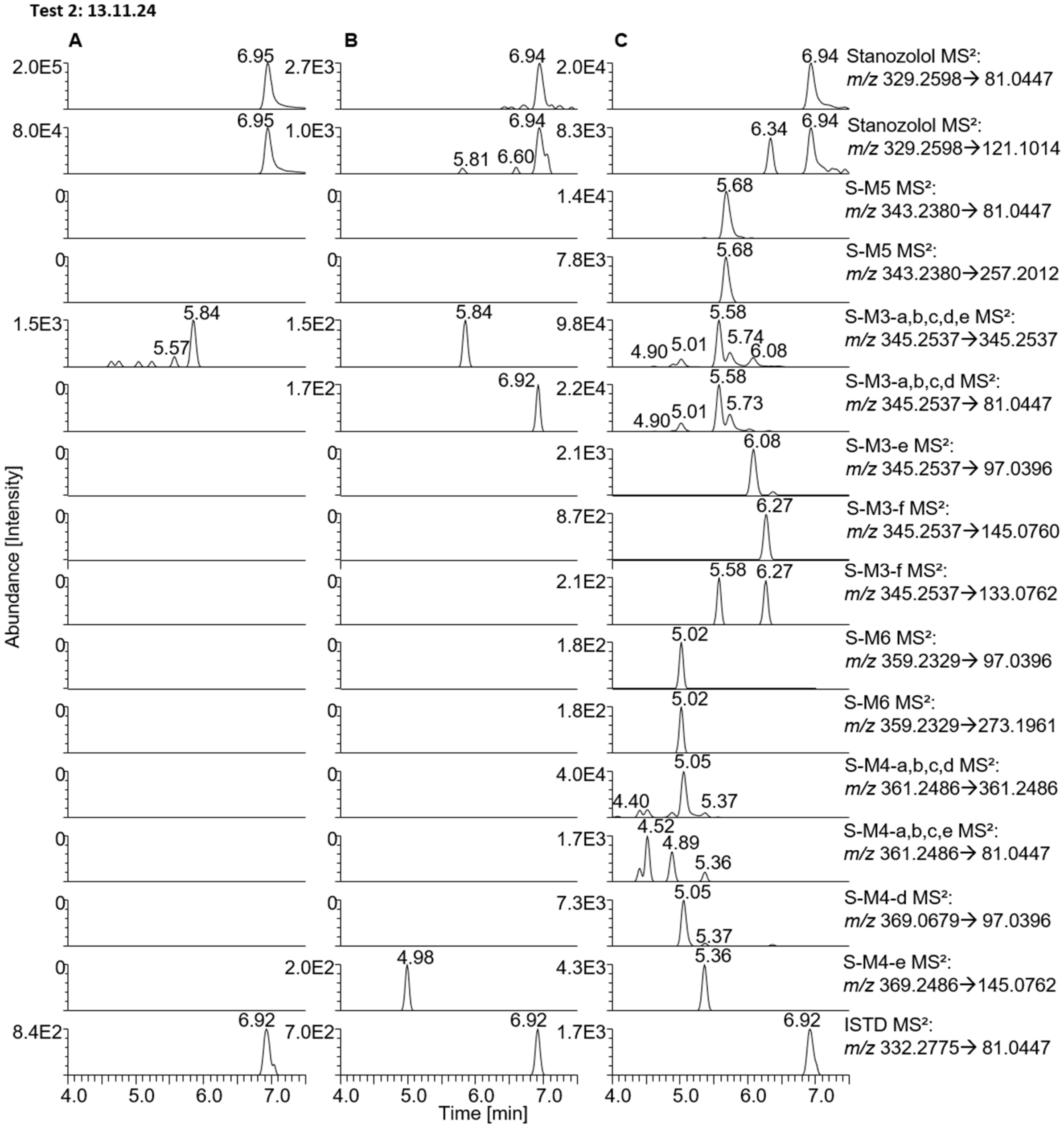

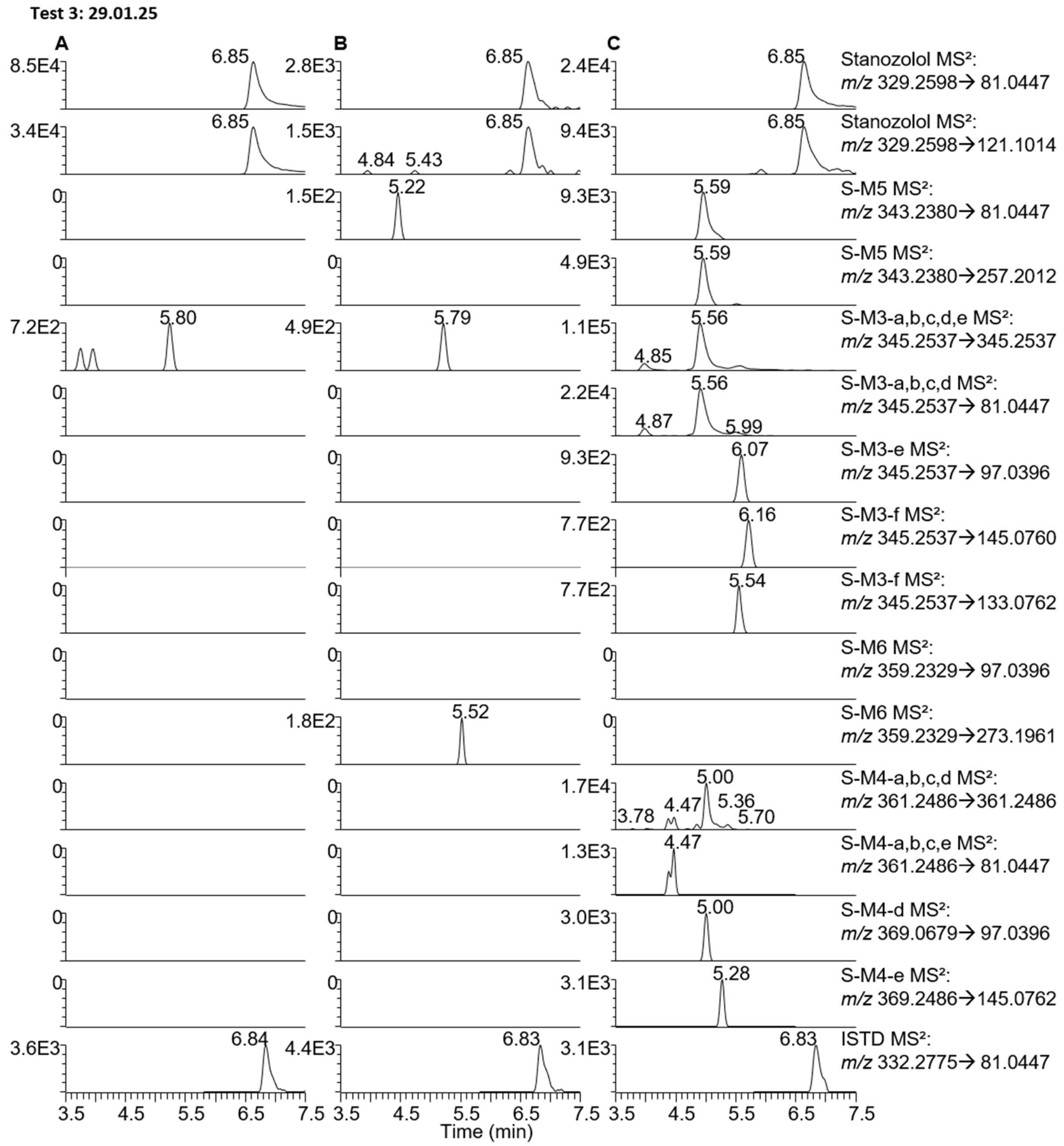

Figure S3: Extracted ion chromatograms of stanozolol (top test 2, bottom test 3) (A) incubated without enzyme, (B) incubated with SV-S9 and (C) with HL-S9 showing the presence of several metabolites only in HL-S9.

4. Extracted ion chromatograms of sample incubated with *GW1516*

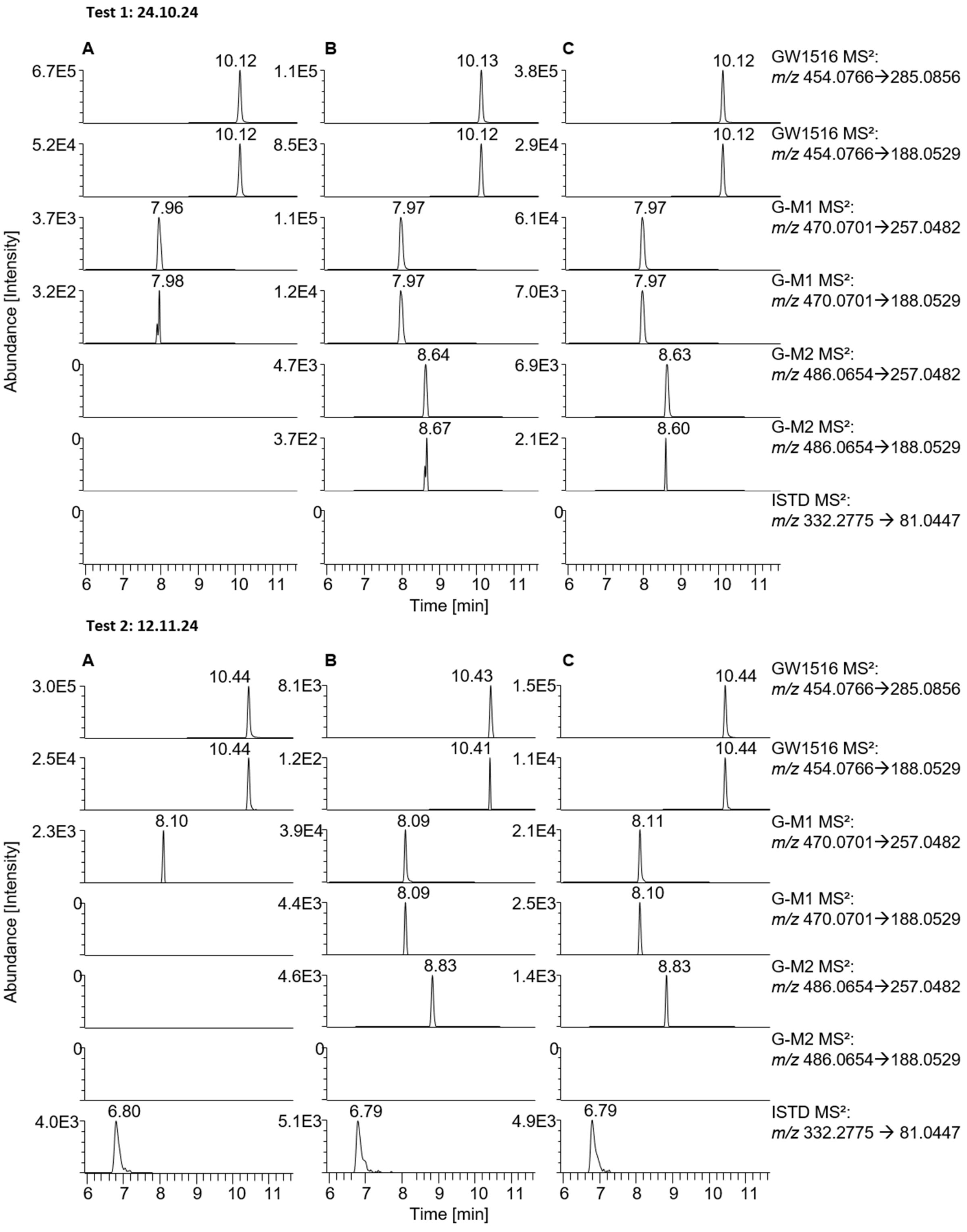

Figure S4: Extracted ion chromatograms of GW1516 (top: test 1, bottom test: 2) (A) incubated without enzyme, (B) incubated with SV-S9 and (C) with HL-S9 showing the presence of several metabolites only in HL-S9.

5. Extracted ion chromatograms of sample incubated with *LGD-4033*

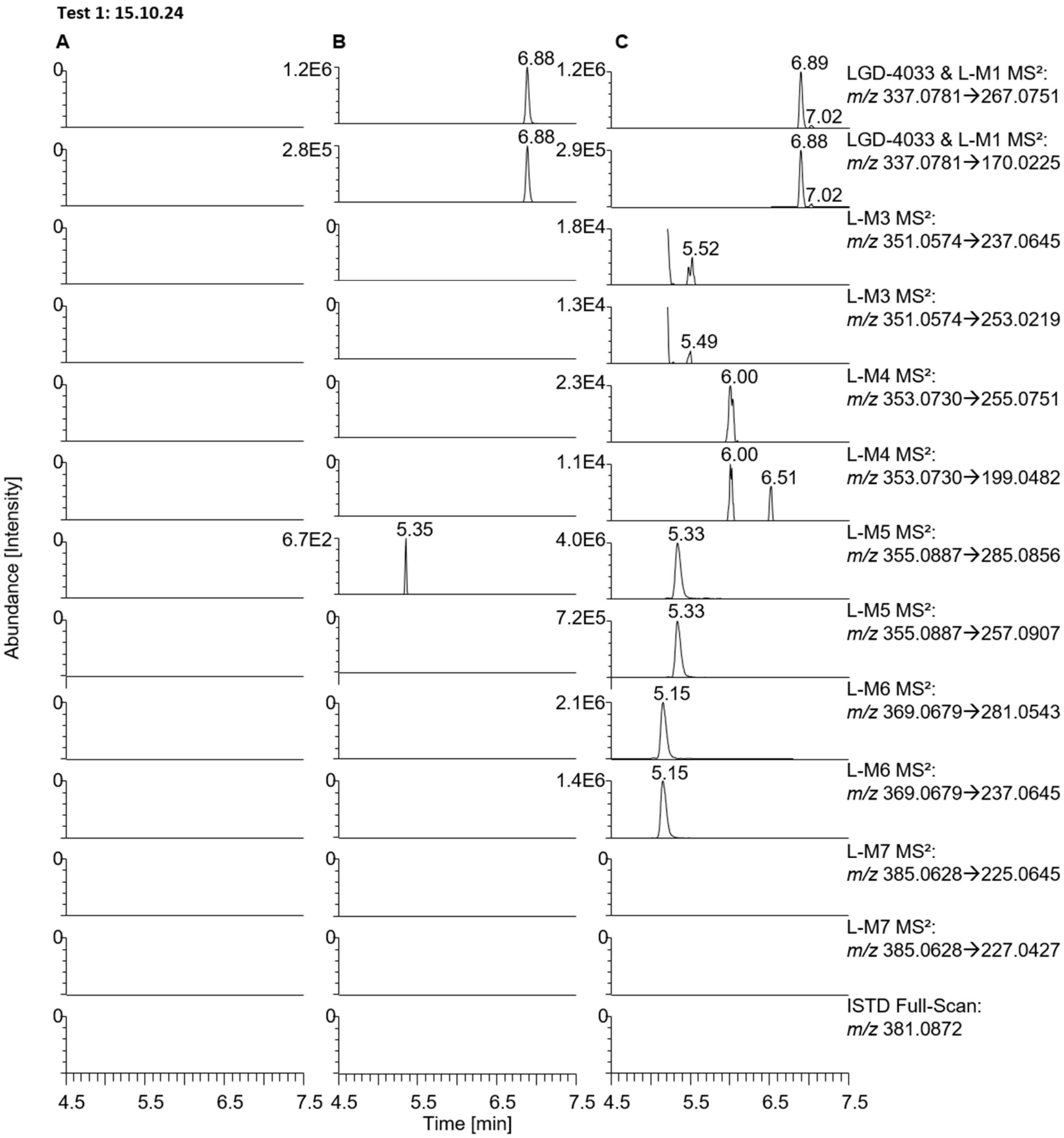

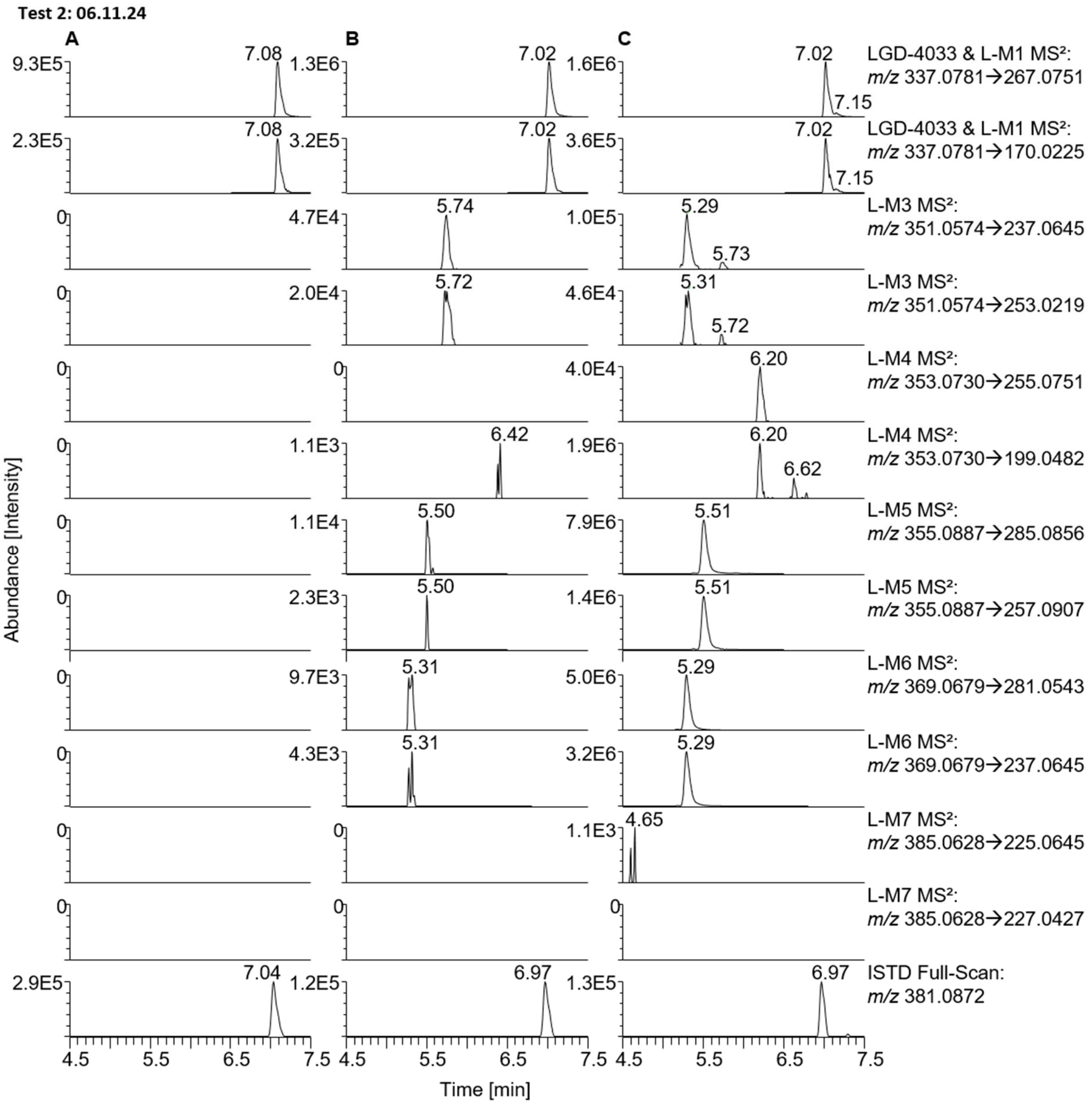

Figure S5 Extracted ion chromatograms of LGD-4033 (top: test 1, bottom test: 2) (A) incubated without enzyme, (B) with SV-S9 and (C) with HL-S9 showing the presence of several metabolites only in HL-S9.

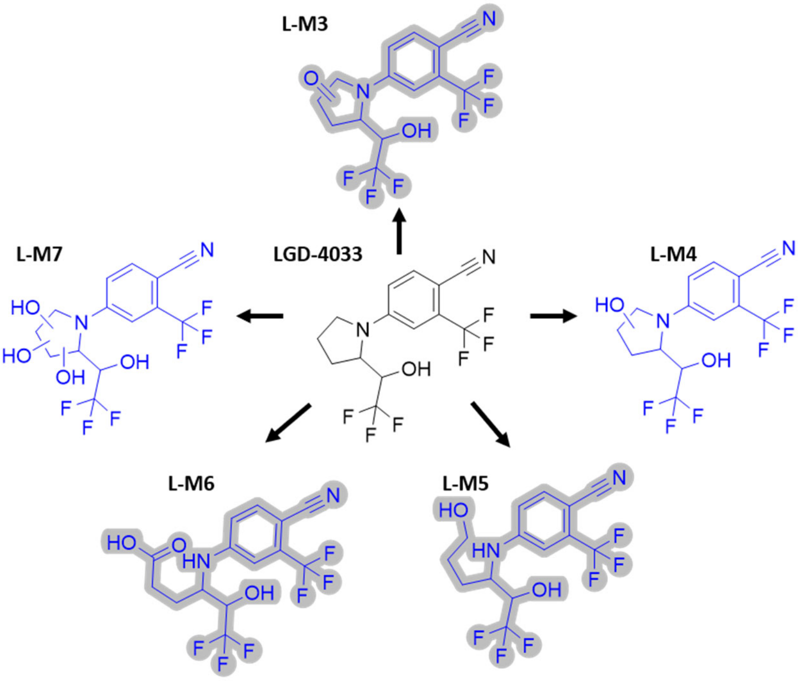

Figure S6 Structures of LGD-4033 and the tentatively identified metabolites *in vitro* using HL-S9 (shown in blue) and SV-S9 (shown in grey) are displayed.

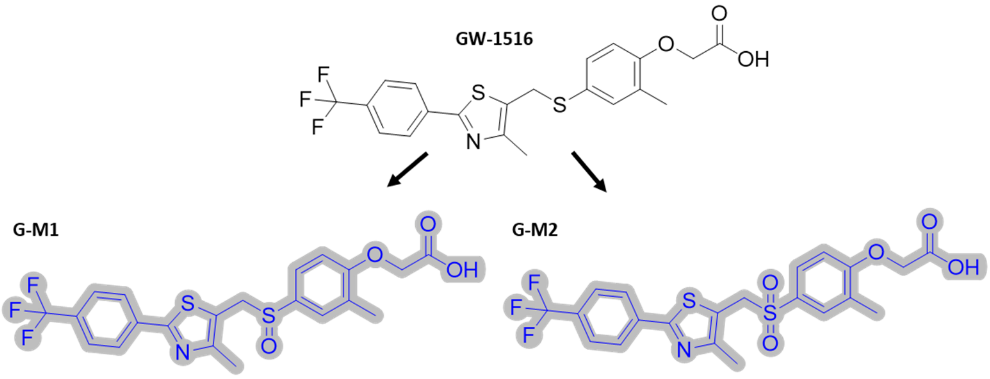

Figure S7 Structures of GW1516 and the tentatively identified metabolites *in vitro* using HL-S9 (shown in blue) and SV-S9 (shown in grey) are displayed.

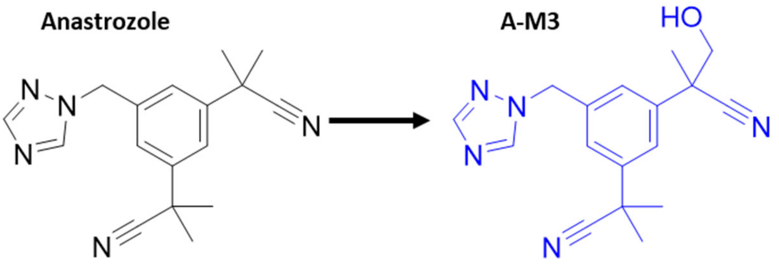

Figure S8 Structures of anastrozole and the tentatively identified metabolite *in vitro* using HL-S9 (shown in blue) are displayed.

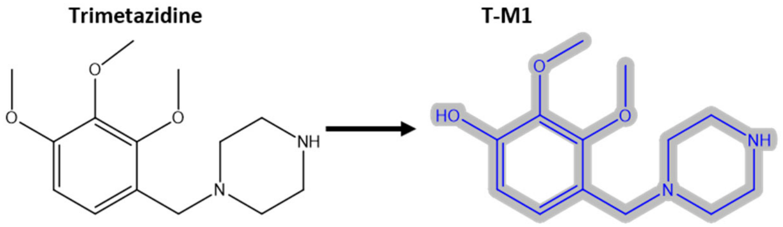

Figure S9 Structures of trimetazidine and the tentatively identified metabolite *in vitro* using HL-S9 (shown in blue) and SV-S9 (shown in grey) are displayed.

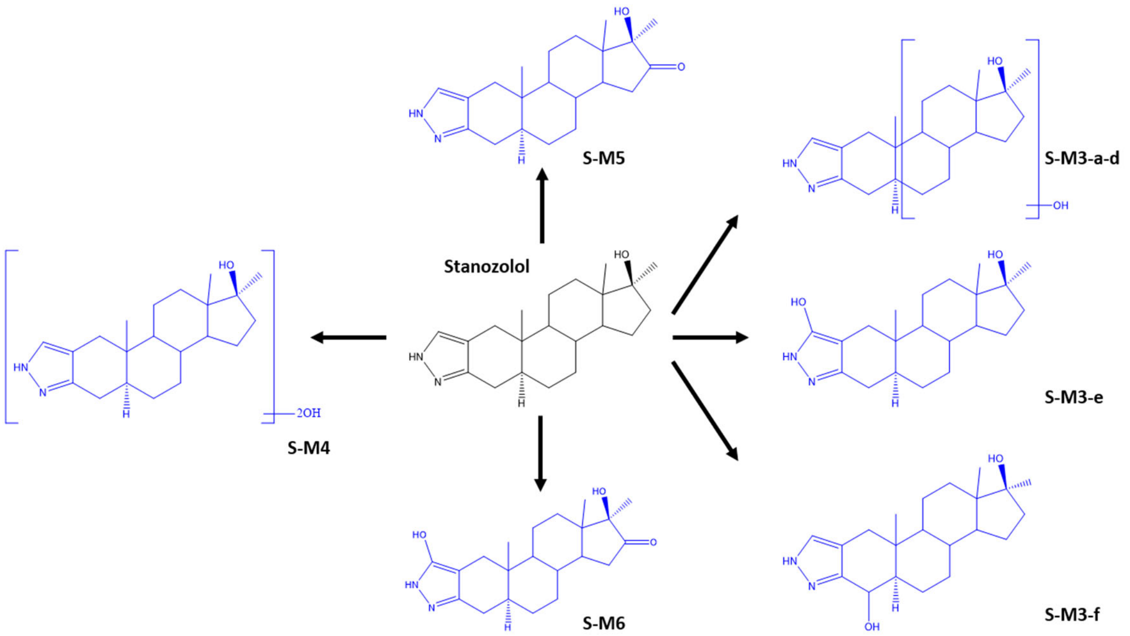

Figure S10 Structures of stanozolol and the tentatively identified metabolites *in vitro* using HL-S9 (shown in blue) are displayed.

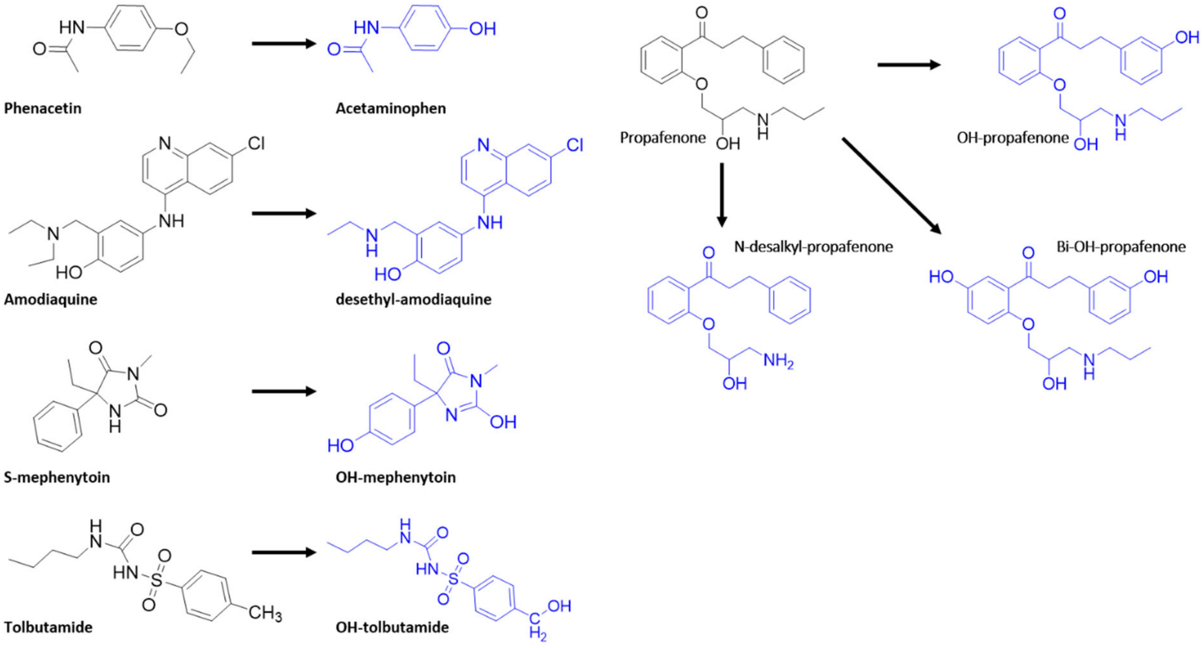

Figure S11 Structures of CYP substrates and the tentatively identified metabolites *in vitro* using HL-S9 (shown in blue) are displayed.

6. **Scatter plots of dPCRs of different CYP enzyme genes**

1-D scatter plots (test 1, test 2) of HL-S9, SV-S9, NTC (non-template control) and PC (positive control) samples for dPCR assays. Each box represents the fluorescence value (expressed as RFU) for each partition, the red line indicates the threshold. Any partition above the threshold (in blue) is considered positive, while any partition below the threshold (in grey) is considered negative.

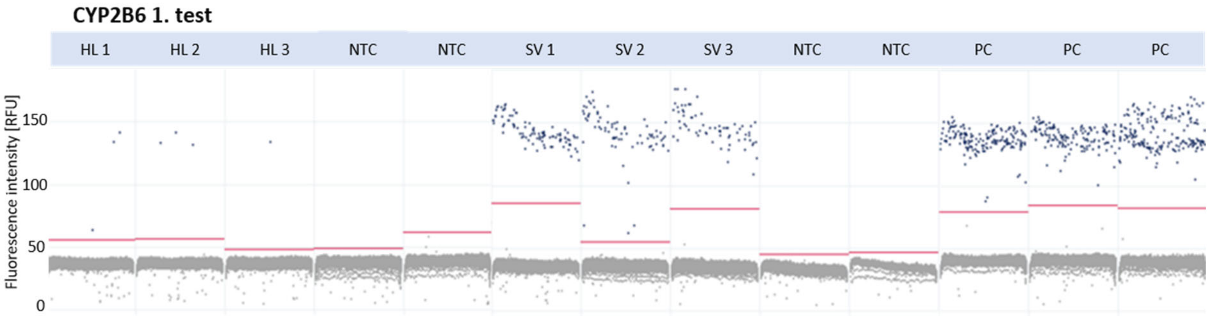

Figure S12 1-D scatter plot (test 1) of HL-S9, SV-S9, NTC and PC samples for dPCR using CYP2B6 primers.

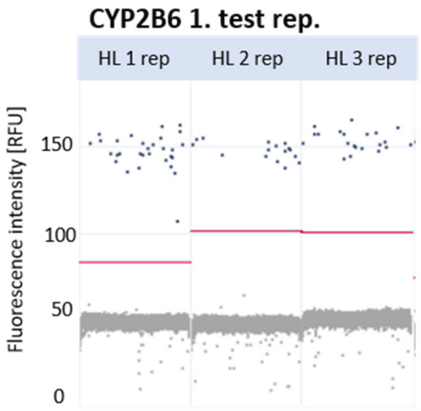

Figure S13 1-D scatter plot (test 1) of a repeat measurement of HL-S9 samples with increased concentration compared to the previous measurement for dPCR using CYP2B6 primers. The adjusted concentration of HL-S9 was used for further measurements.

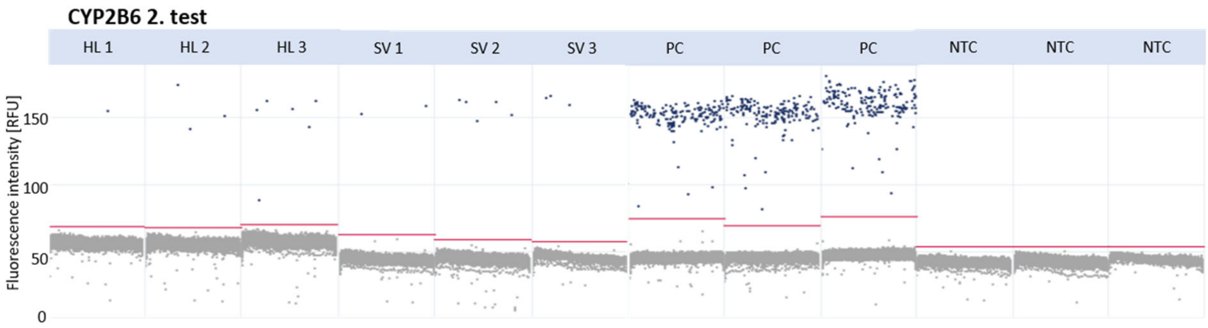

Figure S14 1-D scatter plot (test 2) of HL-S9, SV-S9, NTC and PC samples for dPCR using CYP2B6 primers.

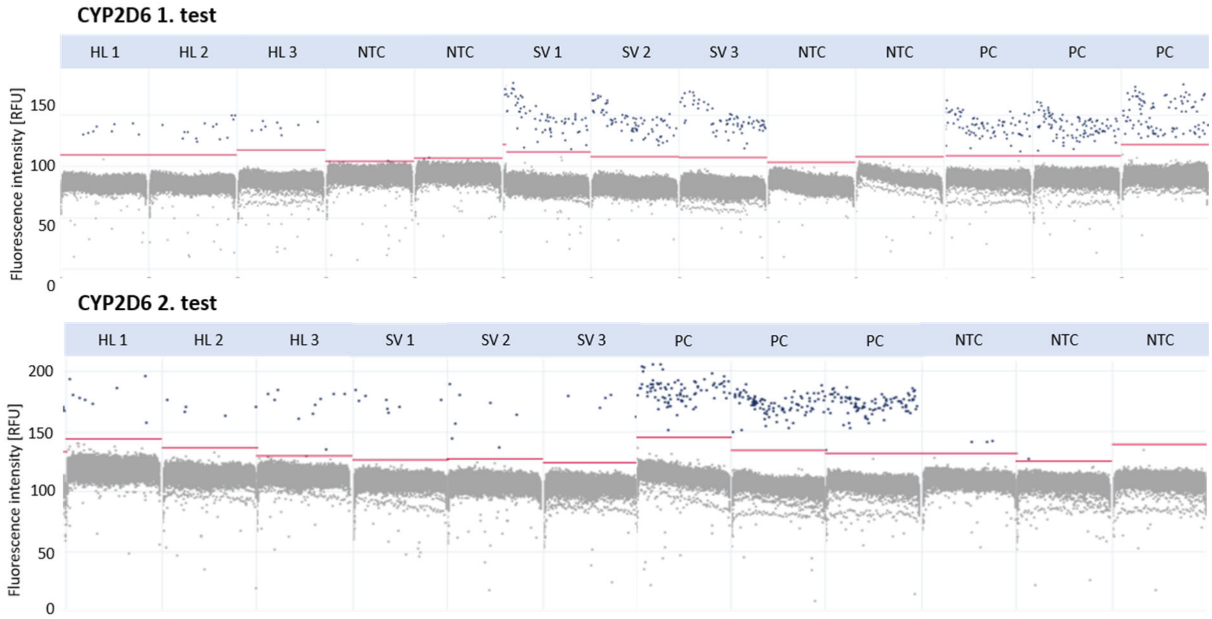

Figure S15 1-D scatter plots (test 1, test 2) of HL-S9, SV-S9, NTC and PC samples for dPCR using CYP2D6 primers.

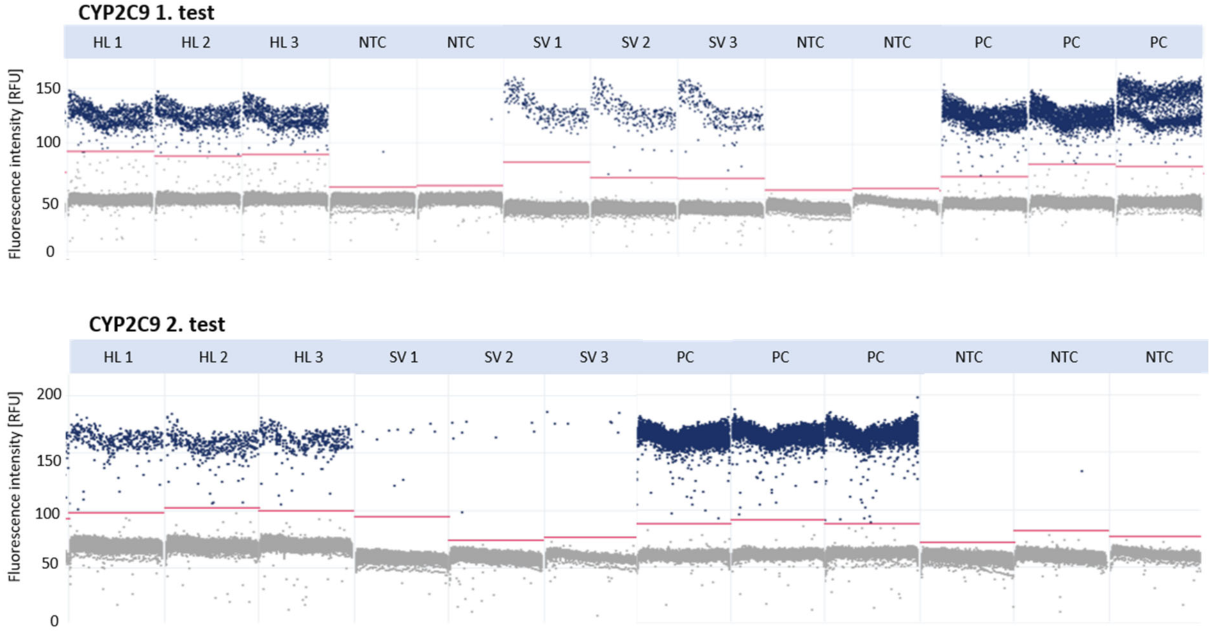

Figure S16 1-D scatter plots (test 1, test 2) of HL-S9, SV-S9, NTC and PC samples for dPCR using CYP2C9 primers.

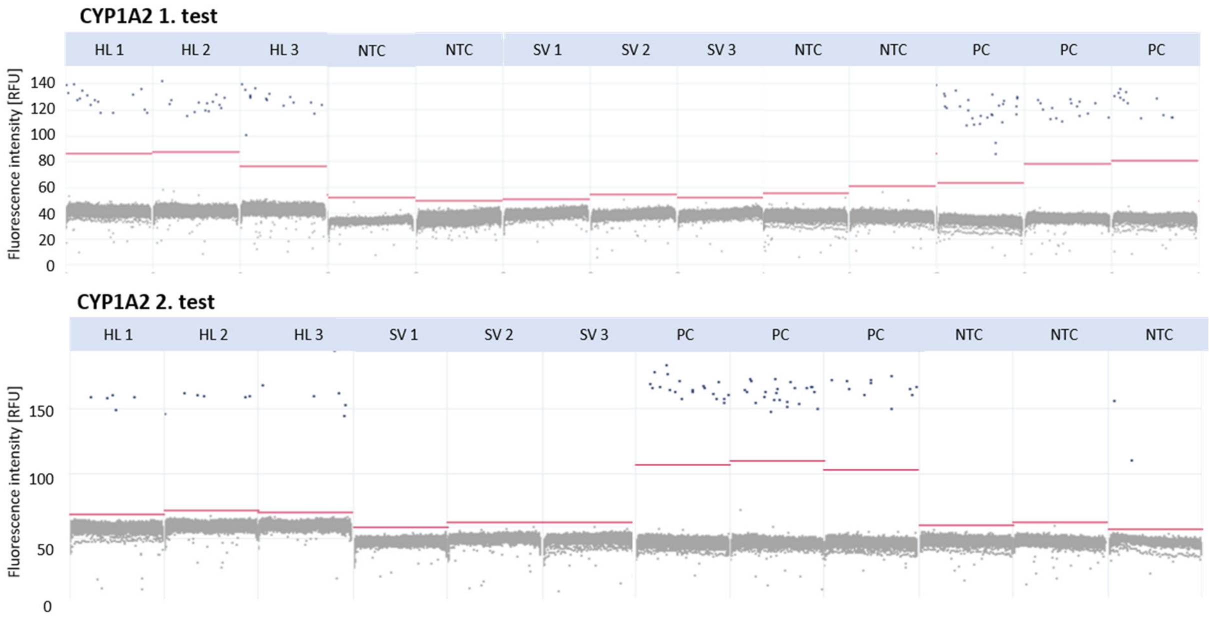

Figure S17 1-D scatter plots (test 1, test 2) of HL-S9, SV-S9, NTC and PC samples for dPCR using CYP1A2 primers.

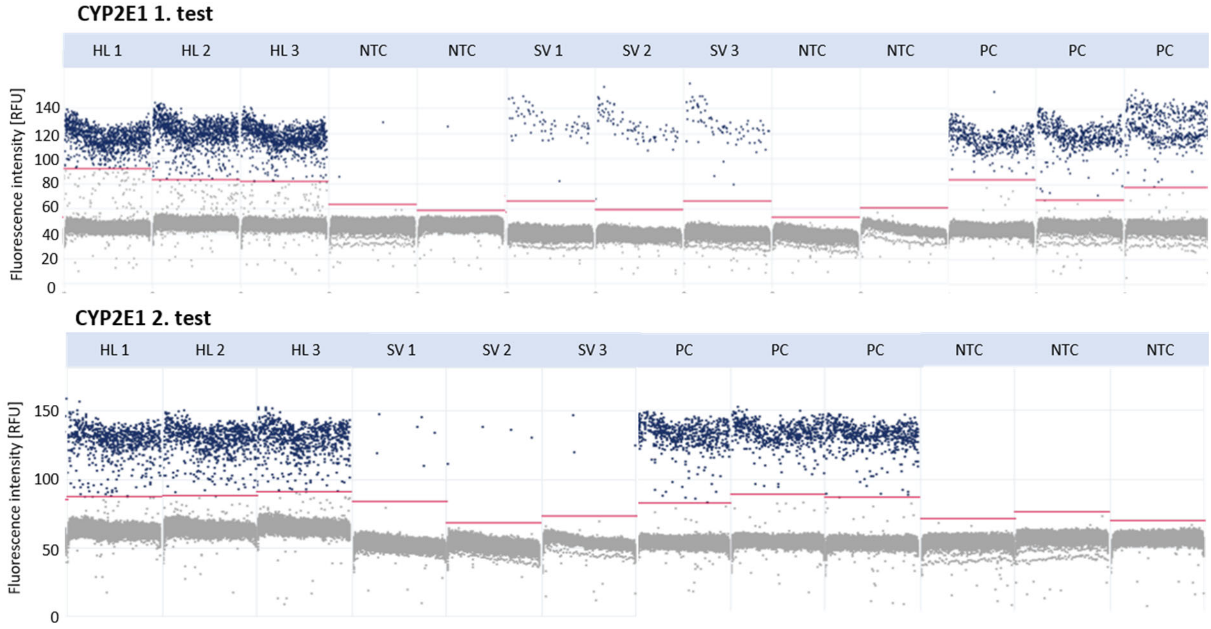

Figure S18 1-D scatter plots (test 1, test 2) of HL-S9, SV-S9, NTC and PC samples for dPCR using CYP2E1 primers.

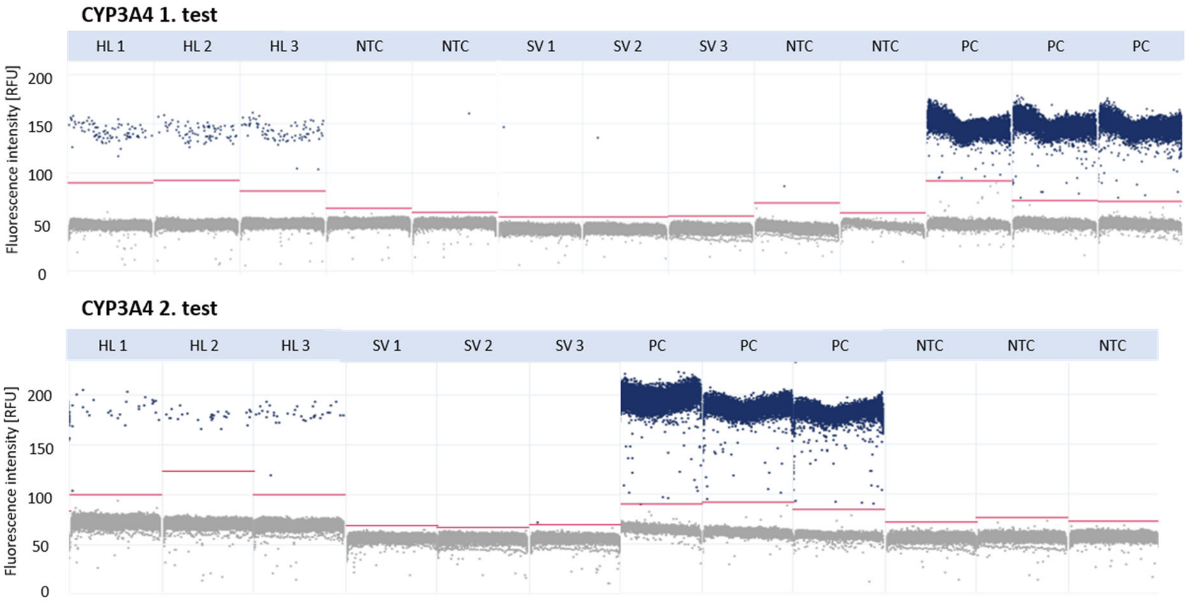

Figure S19 1-D scatter plots (test 1, test 2) of HL-S9, SV-S9, NTC and PC samples for dPCR using CYP3A4 primers.

7. Scatter plots of dPCRs of different Housekeeping genes

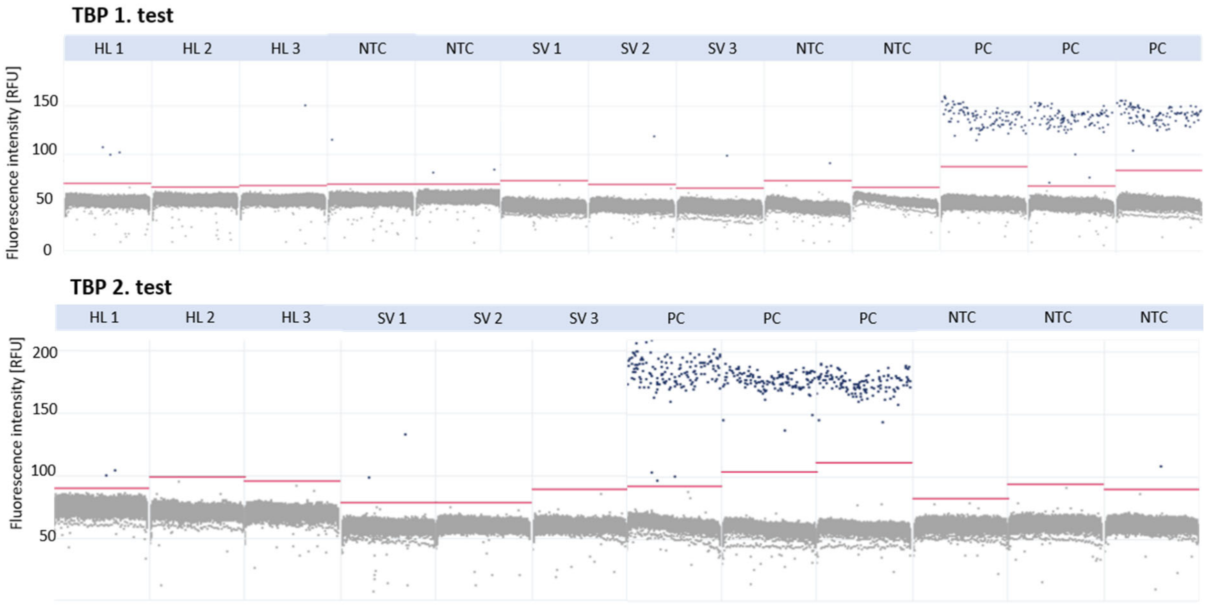

Figure S20 1-D scatter plots (test 1, test 2) of HL-S9, SV-S9, NTC and PC samples for dPCR using TBP primers.

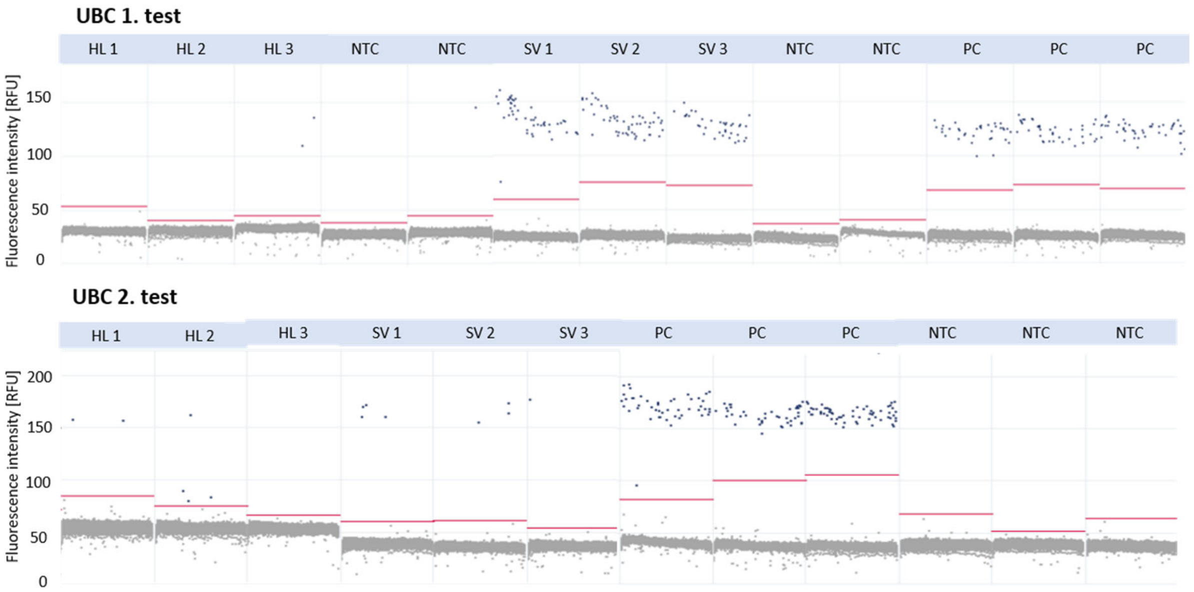

Figure S21 1-D scatter plots (test 1, test 2) of HL-S9, SV-S9, NTC and PC samples for dPCR using UBC primers.

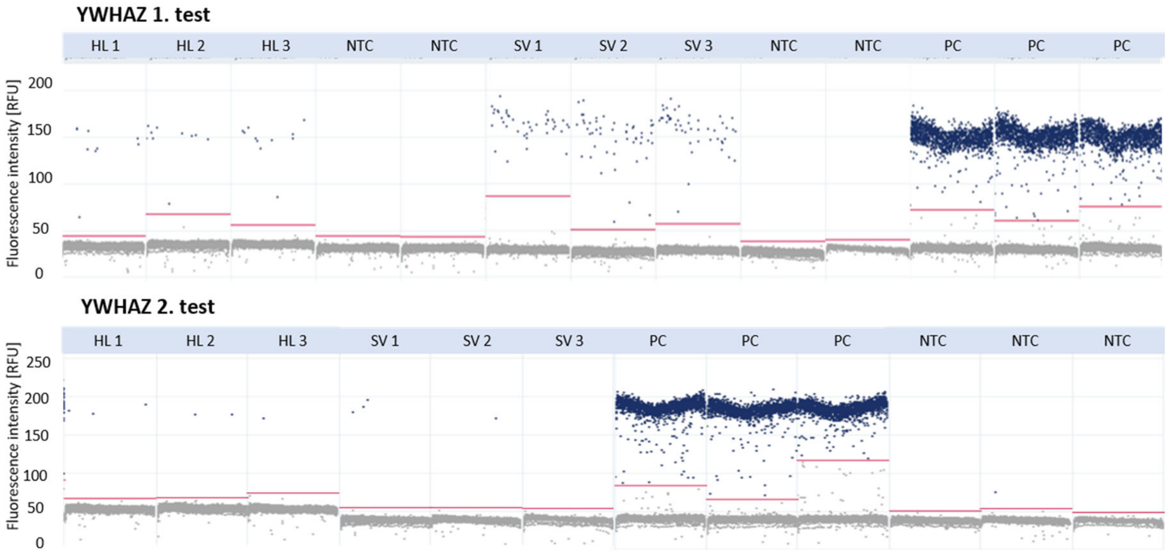

Figure S22 1-D scatter plots (test 1, test 2) of HL-S9, SV-S9, NTC and PC samples for dPCR using YWHAZ primers.
